# Gut microbial metabolite targets HDAC3-FOXK1-interferon axis in fibroblast-like synoviocytes to ameliorate rheumatoid arthritis

**DOI:** 10.1038/s41413-024-00336-6

**Published:** 2024-05-23

**Authors:** Hongzhen Chen, Xuekun Fu, Xiaohao Wu, Junyi Zhao, Fang Qiu, Zhenghong Wang, Zhuqian Wang, Xinxin Chen, Duoli Xie, Jie Huang, Junyu Fan, Xu Yang, Yi Song, Jie Li, Dongyi He, Guozhi Xiao, Aiping Lu, Chao Liang

**Affiliations:** 1 Department of Systems Biology, School of Life Sciences, Southern University of Science and Technology, Guangdong Provincial Key Laboratory of Cell Microenvironment and Disease Research, Shenzhen Key Laboratory of Cell Microenvironment, Shenzhen, 518055 China; 2 Institute of Integrated Bioinfomedicine and Translational Science (IBTS), School of Chinese Medicine, Hong Kong Baptist University, Hong Kong SAR, 999077 China; 3 Department of Biochemistry, School of Medicine, Southern University of Science and Technology, Guangdong Provincial Key Laboratory of Cell Microenvironment and Disease Research, Shenzhen Key Laboratory of Cell Microenvironment, Shenzhen, 518055 China; 4 Division of Immunology and Rheumatology, Stanford University, Stanford, CA 94305 USA; 5 VA Palo Alto Health Care System, Palo Alto, CA 94304 USA; 6 Institute of Plant and Food Science, Department of Biology, Southern University of Science and Technology, Shenzhen, 518055 China; 7 Department of Rheumatology, Guanghua Hospital Affiliated to Shanghai University of Traditional Chinese Medicine, Shanghai University of Traditional Chinese Medicine, Shanghai, China; 8 Department of Computational Biology, St. Jude Children’s Research Hospital, Memphis, TN USA; 9 Department of Laboratory Medicine, Peking University Shenzhen Hospital, Shenzhen, China; 10 Guangdong-Hong Kong-Macau Joint Lab on Chinese Medicine and Immune Disease Research, Guangzhou, 510006 China; 11 Shanghai University of Traditional Chinese Medicine, Shanghai, 200032 China; 12 State Key Laboratory of Proteomics, National Center for Protein Sciences (Beijing), Beijing Institute of Lifeomics, 100850 Beijing, China

**Keywords:** Pathogenesis, Bone

## Abstract

Rheumatoid arthritis (RA) is an autoimmune disease. Early studies hold an opinion that gut microbiota is environmentally acquired and associated with RA susceptibility. However, accumulating evidence demonstrates that genetics also shape the gut microbiota. It is known that some strains of inbred laboratory mice are highly susceptible to collagen-induced arthritis (CIA), while the others are resistant to CIA. Here, we show that transplantation of fecal microbiota of CIA-resistant C57BL/6J mice to CIA-susceptible DBA/1J mice confer CIA resistance in DBA/1J mice. C57BL/6J mice and healthy human individuals have enriched *B. fragilis* than DBA/1J mice and RA patients. Transplantation of *B. fragilis* prevents CIA in DBA/1J mice. We identify that *B. fragilis* mainly produces propionate and C57BL/6J mice and healthy human individuals have higher level of propionate. Fibroblast-like synoviocytes (FLSs) in RA are activated to undergo tumor-like transformation. Propionate disrupts HDAC3-FOXK1 interaction to increase acetylation of FOXK1, resulting in reduced FOXK1 stability, blocked interferon signaling and deactivation of RA-FLSs. We treat CIA mice with propionate and show that propionate attenuates CIA. Moreover, a combination of propionate with anti-TNF etanercept synergistically relieves CIA. These results suggest that *B. fragilis* or propionate could be an alternative or complementary approach to the current therapies.

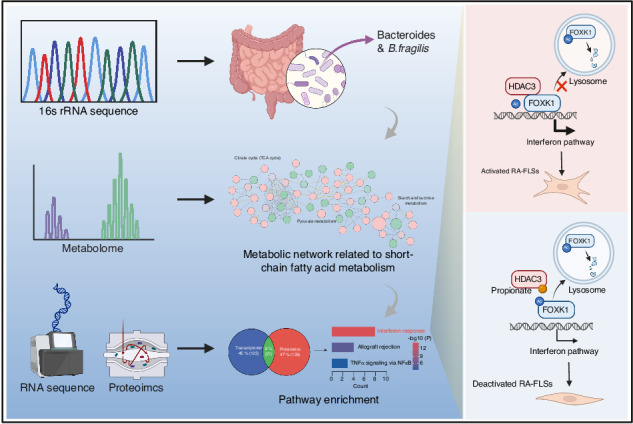

## Introduction

Rheumatoid arthritis (RA) is a systemic and chronic autoimmune disease characterized by inflammation, synovial hyperplasia and destruction of bone and cartilage.^1,2^ More than 0.5%–1.0% of the adult population is affected by RA, with a higher incidence among female and the elderly.^3^ Although the precise cause is not clearly elucidated, it is established that a combination of genetic and environmental factors contribute to the risk of developing RA.^4^ Over the past two decades, immunosuppressive disease-modifying antirheumatic drugs (DMARDs), including conventional synthetic DMARDs (e.g., methotrexate, leflunomide, sulfasalazine, and hydroxychloroquine), biological DMARDs targeting proinflammatory mediators (e.g., TNFα and IL-6) and targeted synthetic DMARDs (e.g., JAK inhibitors), have become the treatment standard for RA.^2,5^ However, high insufficient efficacy leads to relatively low retention rates of them in RA patients, necessitating the exploration of new therapeutic avenues.^6^

Genetically, RA is reported to be a multigene disorder with a heritability estimate of about 65%.^7^ To date, genome-wide association studies have characterized more than a hundred significantly associated loci and thousands of small effect causal variants that contribute to RA susceptibility in different racial and ethnic human populations,^8–11^ at least 30% of which is within the major histocompatibility complex (MHC) genes involved in immunological recognition of self and nonself.^7,12^ These genetic loci are responsible for approximately 15% of the phenotypic variation observed in RA.^13^ However, RA susceptibility still has a large genetic component that is not fully understood.^11^ Ethnically and racially diverse human populations with different genetic frameworks have varied incidences of RA.^14^ The age-adjusted prevalence rates of RA are found to be greater in North America (0.38%), Western Europe (0.35%), and the Caribbean (0.34%) compared to those in Oceania (0.14%) and Western Sub-Saharan Africa (0.13%).^15^ The estimated RA prevalence in Asian regions varies, with South Asia reporting 0.32%, Central Asia 0.21%, East Asia 0.19%, and Southeast Asia showing the lowest at 0.10%.^15^ Currently, advancements in multi-omics data, high-density genotyping, and bioinformatics are empowering researchers to leverage risk variants associated with RA to pinpoint the cell types and biological pathways that play a crucial role in the dysfunctional immune responses and clinical manifestations of the disease.^13^ However, the numerous known genetic variants are far from being employed as therapeutic targets as they are mainly located in non-coding elements that could not be easily manipulated due to potential risks.^16,17^

Over the last few years, gut microbiota has gained attention for their association with RA.^18^ The imbalance of gut microbiota, known as dysbiosis, is proposed to be a significant factor in the development of RA through its influence on the regulation of the host immune system.^19^ Early studies hold an opinion that gut microbiota is environmentally acquired and shaped predominately by extrinsic factors, such as diet, season, smoking and infection.^20,21^ However, accumulating evidence demonstrates that genetic variants also significantly shape the composition of gut microbiota which may then affects the disease susceptibility, suggesting a reciprocal influence between the gut microbiome and the human genome in host immune regulation.^22–25^ Notably, a large-scale population cohort study establish the direct genome-to-genome association between human hosts and gut microbiota and reveals that specific nutritional components determined by the genetic background of hosts can selectively shape the composition of gut microbial strains carrying specific genomic segments.^26^ Genetic variants account for a considerable proportion of variance in gut microbiota in ethnically and racially diverse human populations, with some taxa being 40%.^24^ Thus, we propose the hypothesis that gut microbiota sculpted by genetic factors could potentially predetermine susceptibility to RA prior to the onset of the disease, and genetically resistant RA populations versus genetically susceptible populations could be natural sources for identifying beneficial gut microbiota and developing microbiota-oriented RA therapy.^24^ Disappointingly, the genetics-decided gut microbiota between any two racially different human populations is difficult to be determined by an unbiased approach, as the racially diverse human populations are inevitably exposed to high variability of extrinsic factors that cover up the influence of genetic variants on gut microbiota.^18^

Different mouse strains have disparate genetic backgrounds, which are analogous to the ethnically and racially human populations.^27^ Collagen-induced arthritis (CIA) in mice, which exhibits immunological and pathological characteristics akin to human RA, serves as an optimal model for investigating factors that influence RA susceptibility and for evaluating therapeutic interventions.^28^ It is well known that some strains of inbred laboratory mice (e.g., DBA/1J and B10.RIII) are highly susceptible to CIA, while the others (e.g., C57BL/6J, 129/Sv and BALB/c) are resistant to CIA.^29^ Similar to the role of genetics in human RA, traditional theory holds that genetic variants, especially the MHC genes, dominant the CIA susceptibility among different mouse strains.^30^ Nevertheless, latest studies reveal that genetically diverse mice strains have varied gut microbiota composition even when kept in the same environment, supporting the above opinion in human that genetics shape the gut microbiota.^31,32^ Given the advantage that different mouse strains could be kept and handled identically, we assume that utilization of CIA-susceptible mouse strains versus CIA-resistant mouse strains could provide an alternative option for discovering arthritis-resistant microbes and developing microbiota-oriented treatment options for RA.

In this study, we utilized a CIA-resistant C57BL/6J mouse strain and a CIA-susceptible DBA/1J mouse strain to explore the relationship between gut microbiota and arthritis susceptibility. Transplantation of fecal microbiota of C57BL/6J mice to DBA/1J mice conferred resistance to CIA in DBA/1J mice, driving us to explore the arthritis-resistant gut microbes in C57BL/6J mice. Using 16S ribosomal RNA (rRNA) gene sequencing analysis, we demonstrated that C57BL/6J mice had high abundance of *B. fragilis*, and transplantation of *B. fragilis* prevented CIA in DBA/1J mice. Targeted and untargeted metabolomics revealed that *B. fragilis*-derived propionate was involved in resistance to arthritis. We also showed that abundance of *B. fragilis* and level of propionate were higher in healthy individuals than those in RA patients. Propionate, belonging to the class of short-chain fatty acids (SCFAs), is a metabolite derived from the fermentation of dietary fiber by commensal bacteria.^33^ Propionate has been recognized for their role in regulating immune cell function in autoimmune diseases.^33^ However, the impact of SCFAs on structural cells of joints remains poorly understood. Fibroblast-like synoviocytes (FLSs) are important structural cells and can be found in both the lining and the sublining layers of the synovial membrane of joint.^34^ They are essential for maintaining the homeostasis of joints, where they product nutritive plasma and extracellular matrix containing lubricating molecules such as hyaluronic acid.^34^ In RA, FLSs are activated to undergo phenotypic transformation into tumor-like cells and also produce a lot of inflammatory mediators, leading to growing synovial pannus that invades adjacent cartilage and bone.^35,36^ We examined the effects of propionate on RA-FLSs and demonstrated that propionate inhibited pathological phenotypes of RA-FLSs in vitro. Moreover, we conducted in vivo studies and showed that propionate monotherapy or a combination of propionate with an anti-TNF DMARD, achieved desirable therapeutic efficacy for arthritis.

To determine the mechanism of action of propionate, we performed transcriptomics and proteomics and found that propionate mainly blocked the interferon pathway in RA-FLSs. Among the differentially expressed proteins in RA-FLSs treated with propionate, Forkhead Box K1 (FOXK1), attracted out attention as it was a transcription factor at the upstream of interferon pathway.^37^ FOXK1 was downregulated at protein level but not at mRNA level by propionate in RA-FLSs. To date, most studies related to FOXK1 emphasize its critical role in metabolic regulation, such as the glucose and lipid metabolism.^38,39^ Role of FOXK1 in RA-FLSs are not well understood. We revealed that gene knockdown of FOXK1 inhibited interferon signaling and pathological transformation of RA-FLSs, suggesting that FOXK1 a potential target in RA-FLSs. To explain how propionate modulated FOXK1-interferon pathway, we examined activity of histone deacetylases (HDACs), as propionate has been proven to be an inhibitor of HDACs, especially HDAC3.^40^ HDACs are important epigenetic regulators.^41^ They function as enzymes, catalyzing the detachment of acetyl groups from the lysine residues on numerous proteins, consequently impacting protein stability at the post-translational levels.^42^ We found that FOXK1 served as another substrate of HDAC3 in RA-FLSs and interaction between HDAC3 and FOXK1 is required for protecting FOXK1 from lysosomal system-mediated degradation. Propionate disrupted HDAC3-FOXK1 interaction to increase acetylation of FOXK1, resulting in reduced FOXK1 stability, blocked interferon signaling and deactivation of RA-FLSs.

This study establishes the connection between gut microbiota and FLSs in CIA mice and RA patients. *B. fragilis* exhibits functional effects on RA-FLS via producing propionate, which targets HDAC3-FOXK1-interferon axis to inhibit tumor-like transformation of RA-FLSs. *B. fragilis*-oriented therapy or propionate could be an alternative or complementary treatment for RA to the current DMARDs.

## Results

### Fecal microbiota of C57BL/6J mice conferred resistance to CIA in DBA/1J mice

**Fig. 1:**
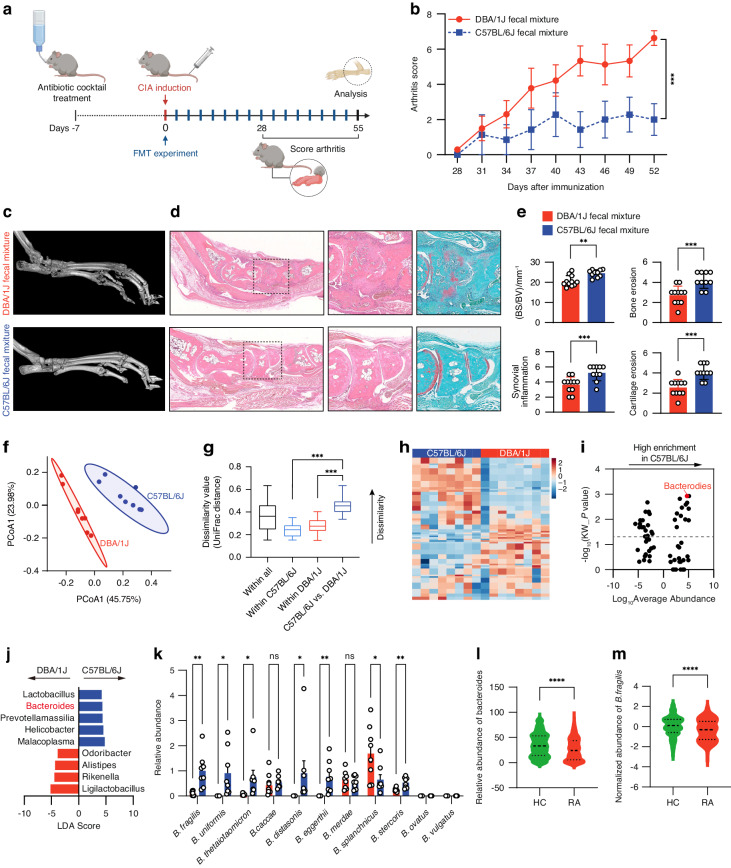
Fecal microbiota transplantation and differential composition of gut microbiota between donor C57BL/6J mice and recipient DBA/1J mice. **a** Illustration of the fecal microbiota transplantation (FMT) in DBA1/J mice prior to the onset of CIA. **b** Evaluation of arthritis scores for the mice after FMT. **c** Representative micro-CT images of the mice after FMT. **d** Images of H&E and SafO-FG staining of the paw sections. **e** Measurement of ratio of bone surface to bone volume (BS/BV), and quantification of synovial inflammation and bone erosion on H&E-stained sections, as well as cartilage erosion on SafO-FG-stained sections. *n* = 10 for mice treated with C57BL/6J fecal mixture and *n* = 11 for mice treated with DBA/1J fecal mixture. **f** Principal coordinate analysis (PCoA) plots of 16S rRNA gene sequencing data depicting the differential microbial composition between non-immunized C57BL/6J mice and DBA/1J mice. **g** Boxplots showing unweighted UniFrac distances within and between samples. **h** Heatmap of bacterial operational taxonomic units (OTUs) abundance in non-immunized DBA/1J mice and C57BL/6J mice. **i** Volcano plot displaying the differential abundance distribution of microbial OTUs at the genus level in non-immunized C57BL/6J mice. The horizontal dotted line showed the *P* value = 0.05, and the OTUs above horizontal dotted line had statistical difference between the two mouse strains. **j** Linear discriminant analysis (LDA) effect size analysis of differential microbial abundance at the genus level between non-immunized DBA/1J mice and C57BL/6J mice with an LDA score >3. **k** Relative abundance of human-related species of Bacteroides in feces of non-immunized C57BL/6 mice and DBA/1J mice as detected by real-time PCR. *n* = 8 for both DBA/1J mice and C57BL/6J mice. **l** Relative abundance of Bacteroides in feces of RA patients (*n* = 222) and healthy control (HC) individuals (*n* = 1 000) from GMrepo Database. **m** Normalized abundance of *B.fragilis* in feces of RA patients (*n* = 143) and HC individuals (*n* = 1 000) from GMrepo Database. Graphs represented means ± SEM and statistical significance was calculated by two-way analysis of variance (ANOVA) (**b**), Student’s *t* test (**e** and **k**–**m**) and one-way ANOVA (**g**). ns no significance, *P < 0.05, **P < 0.01, ***P < 0.001 and ****P < 0.000 1

We performed CIA induction to confirm the distinct susceptibility to CIA between the C57BL/6J mice and the DBA/1J mice in our housing environment (Fig. S1A). After immunization with type II collagen, we observed that C57BL/6J mice barely exhibited arthritis symptoms, whereas DBA/1J mice displayed a very high arthritic score, and severe bone erosion, synovial inflammation and cartilage erosion (Fig. S1B–E). To examine whether the C57BL/6J mice had arthritis-resistant gut microbiota, we collected fecal samples of non-immunized C57BL/6J mice and DBA/1J mice and performed fecal microbiota transplantation (FMT) experiment in DBA/1J mice prior to the onset of CIA (Fig. 1a). The DBA/1J mice receiving the fecal mixture from donor C57BL/6J mice showed lower arthritic score when compared to the mice receiving the fecal mixture from donor DBA/1J mice (Fig. 1b). Micro-CT and histological examination by hematoxylin and eosin (H&E) and Safranin O & Fast Green (SafO-FG) staining showed that the DBA/1J mice receiving the fecal mixture from donor C57BL/6J mice had attenuated bone erosion, synovial inflammation and cartilage erosion (Fig. 1c, d). Quantification of ratio of bone surface to bone volume (BS/BV), bone erosion, synovial inflammation and cartilage erosion consistently demonstrated that the FMT between donor C57BL/6J mice and recipient DBA/1J mice conferred resistance to CIA (Fig. 1e).

### Differential gut microbiota composition between C57BL/6J and DBA/1J mice

We conducted a high-throughput 16S rRNA gene sequencing analysis using fecal samples from both non-immunized DBA/1J mice and C57BL/6J mice. Beta diversity of microbial communities was analyzed by principal coordinate analysis (PCoA) based on unweighted UniFrac distances. There was a clear separation of microbial composition between DBA/1J and C57BL/6J mice (Fig. 1f). The gut microbiota composition exhibited a statistically significant variation between the DBA/1J and C57BL/6J mouse strains (Fig. 1g). Heatmap analysis displayed 91 bacterial operational taxonomic units (OTUs) in total that were detected in both DBA/1J mice and C57BL/6J mice (Fig. 1h). Volcano plot showed the differentially abundant OTUs at the genus level between the two mouse stains, among which Bacteroides was the most enriched genus with the lowest P value in C57BL/6J mice (Fig. 1i). Then, linear discriminant analysis (LDA) effect size analysis was performed and showed that five taxa were enriched with an LDA score >3 in C57BL/6J mice, which also included the Bacteroides (Fig. 1j). We also performed 16S rRNA gene sequencing using fecal samples from both DBA/1J and C57BL/6J mice with established CIA (Fig. S2A). We observed the differential gut microbiota composition between the two mouse strains (Fig. S2B, C). Relative abundance of Bacteroides was also higher in C57BL/6J mice than that in DBA/1J mice (Fig. S2D). To date, more than 30 species of Bacteroides have been recognized, among which *B. fragilis, B. vulgatus*, *B. stercoris*, *B. eggerthii*, *B. uniformis*, *B. caccae*, *B. thetaiotaomicron*, *B. ovatus*, *B. splanchnicus*, *B. merdae*, and *B. distasonis* have been detected in human samples.^43–45^ We analyzed these species in the non-immunized DBA/1J mice and C57BL/6J mice and verified that *B. fragilis*, *B. eggerthii and B.stercoris* were more significantly enriched than other species in the fecal samples of C57BL/6J mice (Fig. 1k). Previous studies have reported that mice gavaged with *B. eggerthii* may develop more severe colitis, and *B. stercoris* was associate with a higher risk to develop colorectal cancer.^46,47^ Thus, we focused on *B. fragilis* for further studies. The fecal abundance of *B. fragilis* was also higher in C57BL/6J mice than that in DBA/1J mice with established CIA (Fig. S2E). In addition, we also compared the fecal abundance of Bacteroides and *B. fragilis* between RA patients and healthy control (HC) individuals in a GMrepo Database.^48^ RA patients had lower level of Bacteroides and *B. fragilis* than HC individuals (Fig. 1l, m). These results suggested that *B. fragilis* might be associated with the resistance to arthritis.

### *B. fragilis* transplantation ameliorated arthritis in DBA/1J mice

**Fig. 2:**
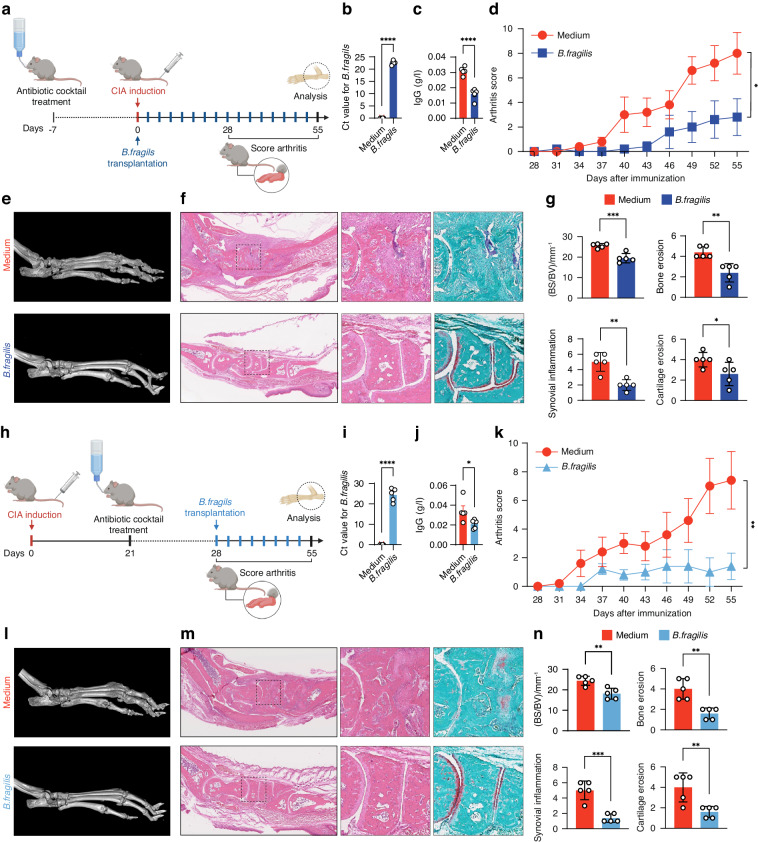
Effects of B. fragilis transplantation on arthritis development in DBA/1J mice. **a** Illustration of *B. fragilis* transplantation in DBA/1J mice prior to the onset of CIA. Briefly, DBA1/J mice were pre-treated with antibiotic cocktail for one week and then immunized with type II collagen to induce CIA. With the beginning of CIA induction, the mice were orally administrated with either *B. fragilis* or medium twice a week for eight weeks. **b** The colonization efficacy of *B. fragilis* in DBA/1J mice after one week of *B. fragilis* transplantation as detected by real-time PCR. **c** Level of serum lgG in the mice after *B. fragilis* transplantation as detected by an automated hematology analyzer. **d** Evaluation of arthritis scores for the mice after *B. fragilis* transplantation. **e** Representative micro-CT images of paws of the mice after *B. fragilis* transplantation. **f** Images of H&E and SafO-FG staining of the paw sections. **g** Measurement of BS/BV, and quantification of synovial inflammation and bone erosion on H&E-stained sections, as well as cartilage erosion on SafO-FG-stained sections. **h** Levels of serum lgG in the mice after *B. fragilis* transplantation as detected by an automated hematology analyzer. **i** Illustration of *B. fragilis* transplantation in DBA/1J mice with established CIA. DBA1/J mice were immunized with type II collagen for four weeks to establish CIA. During the last week of CIA establishment, the mice were pre-treated with antibiotic cocktail and then orally administrated with either *B. fragilis* or medium control for another four weeks. **j** The colonization efficacy of *B. fragilis* in DBA/1J mice with established CIA after one week of *B. fragilis* transplantation. **k** Evaluation of arthritis scores for the mice with established CIA after *B. fragilis* transplantation. **l** Representative micro-CT images of the mice with established CIA after *B. fragilis* transplantation. **m** Images of H&E and SafO-FG staining of the paw sections. **n** Measurement of BS/BV, and quantification of synovial inflammation and bone erosion on H&E-stained sections, as well as cartilage erosion on SafO-FG-stained sections of paws of the mice with established CIA after *B. fragilis* transplantation. *n* = 5 for each treatment group. Graphs represented means ± SEM and statistical significance was calculated by two-way ANOVA (**d** and **k**) and Student’s *t* test (**b**, **c**, **g**, **i**, **j** and **n**). *P < 0.05, **P < 0.01, ***P < 0.001 and ****P < 0.000 1

We assessed the protective potential of *B. fragilis* transplantation against CIA. DBA/1J mice were orally administered with *B. fragilis* or medium control prior to the onset of CIA (Fig. 2a). The colonization efficacy was confirmed after administration of *B. fragilis* (Fig. 2b). Transplantation of *B. fragilis* significantly reduced serum lgG and arthritic scores in DBA/1J mice when compared to the medium control (Fig. 2c, d). Micro-CT and histological examination showed that administration of *B. fragilis* ameliorated bone erosion, synovial inflammation and cartilage erosion in DBA/1J mice (Fig. 2e, f). Quantification of BS/BV, bone erosion, synovial inflammation and cartilage erosion confirmed the protective action of *B. fragilis* against arthritis in DBA/1J mice (Fig. 2g). Furthermore, we also evaluated the therapeutic potential of *B. fragilis* transplantation in DBA/1J mice with established CIA. After type II collagen immunization, DBA/1J mice exhibiting arthritis symptoms were orally administered with *B. fragilis* or medium control (Fig. 2h). We observed the efficient colonization of *B. fragilis* in the CIA mice (Fig. 2i). Transplantation B. *fragilis* resulted in lower serum lgG and arthritic scores (Fig. 2j, k) and inhibited bone erosion, synovial inflammation and cartilage erosion in the CIA mice (Fig. 2l–n). These results indicated that *B. fragilis* could be a beneficial gut bacterial against arthritis occurrence and development.

### *B.fragilis*-derived propionate was involved in resistance to arthritis

**Fig. 3:**
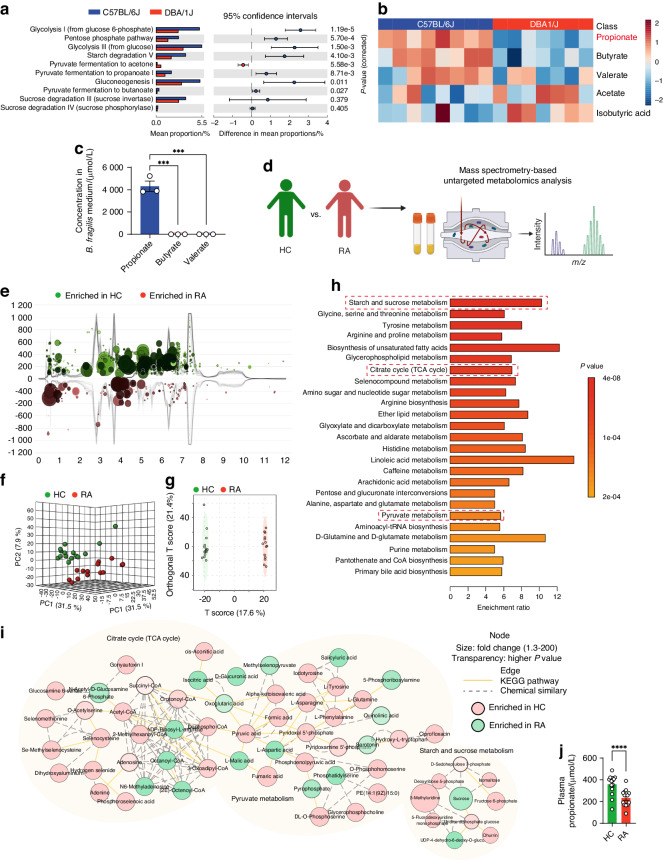
Prediction of gut microbial function profiles and metabolomic analysis of human plasma. **a** Prediction of functional profiles based on 16S rRNA gene sequencing data of C57BL/6J mice and DBA1/J mice using the Phylogenetic Investigation of Communities by Reconstruction of Unobserved States (PICRUSt2) software and the MetaCyc database. Extended error bar plots were created by the STAMP software. **b** Heatmap showing the relative levels of fecal short-chain fatty acids (SCFAs) as detected by gas chromatography-mass spectrometry (GC-MS). *n* = 8 for C57BL/6J mice and *n* = 7 for DBA/1J mice. **c** Levels of propionate, butyrate and valerate in the medium fermented by *B. fragilis*, as detected by GC-MS. Experiments were performed in triplicate and repeated three times. **d** Illustration of untargeted plasma metabolomics in RA patients and HC individuals. **e** Metabolomic total ion chromatogram cloudplot of significant metabolite features in plasma between RA patients and HC individuals. Each metabolite feature was represented by a bubble. The fold change was used as radius scale of each bubble. Darker color (in red and/or in blue tones) of the bubble indicated lower *P* value. **f** Score plots of three-dimension principal component analysis (3D-PCA) for metabolic profiles between RA patients and HC individuals. **g** Score plots of orthogonal partial least squares discriminant analysis (OPLS-DA) based on plasma metabolic profiles of RA patients and HC individuals. **h** Metabolite set enrichment analysis (MSEA) of downregulated pathways in RA patients based on significantly altered metabolites mapped in KEGG human metabolic pathways. **i** Metabolic network integrated with biochemical pathways and chemical relationships of TCA cycle, pyruvate metabolism and starch and sucrose metabolism. **j** Plasma level of propionate in RA patients and HC individuals as measured by GC-MS. *n* = 14 for RA patients and *n* = 15 for HC individuals. Graphs represented means ± SEM and statistical significance was calculated by one-way ANOVA (**c**) and Student’s *t* test (**j**). ***P < 0.001 and ****P < 0.000 1

Bacteroides produce various SCFAs by fermentation of dietary polysaccharides.^49–51^ Using the Picrust2 software and the MetaCyc database, we conducted 16S rRNA gene sequencing-based prediction of functional profiles associated with SCFAs metabolism. There was more significant enrichment of metabolic pathways related to SCFAs production in the C57BL/6J mice when compared to the DBA/1J mice, such as glycolysis, starch degradation, pyruvate fermentation to propanoate and sucrose degradation (Fig. 3a). Levels of SCFAs, including propionate, butyrate, valerate, acetate and isobutyric acid, were analyzed in fecal samples of the C57BL/6J and DBA/1J mice using propyl chloroformate (PCF) derivatization followed by gas chromatography-mass spectrometry (GC-MS) (Fig. S3A–D). C57BL/6J mice had higher levels of propionate, butyrate and valerate rather than acetate and isobutyric acid when compared to DBA/1J mice (Fig. 3b). As *B. fragilis* is a dominant species of Bacteroides in C57BL/6J mice and *B. fragilis* transplantation ameliorated arthritis in DBA/1J mice, we detected the levels of propionate, butyrate and valerate in the medium fermented by *B. fragilis* and observed that *B. fragilis* produced more propionate than butyrate and valerate (Fig. 3c), suggesting that propionate produced by *B. fragilis* might be involved in resistance to arthritis.

We also collected plasma samples of RA and HC individuals and performed untargeted metabolomic analysis (Fig. 3d). The metabolomic total ion chromatogram cloudplot, score plots of three-dimension principal component analysis (3D-PCA), and score plots of orthogonal partial least squares discriminant analysis (OPLS-DA) revealed that RA had distinct metabolite profiles with HC individuals (Fig. 3e–g). There was a variety of significantly changed metabolites between RA patients and HC individuals (Fig. S4). Metabolite set enrichment analysis (MSEA) showed that SCFAs-related metabolic pathways, such as the TCA cycle, pyruvate metabolism, and starch and sucrose metabolism, were significantly downregulated in RA patients when compared to those of HC individuals (Fig. 3h). Metabolic network integrated with biochemical pathways and chemical relationships indicated the significant enrichment of TCA cycle, pyruvate metabolism and starch and sucrose metabolism in HC individuals rather than in RA patients (Fig. 3i). Moreover, we detected the level of propionate in plasma samples using targeted metabolomics and observed decreased propionate in RA patients than that in HC individuals (Fig. 3j). These results suggested that the SCFAs-related metabolic pathways are abnormal in RA patients, leading to less production of propionate and loss of resistance to arthritis.

### Propionate inhibited pathological phenotypes of RA-FLSs in vitro

**Fig. 4:**
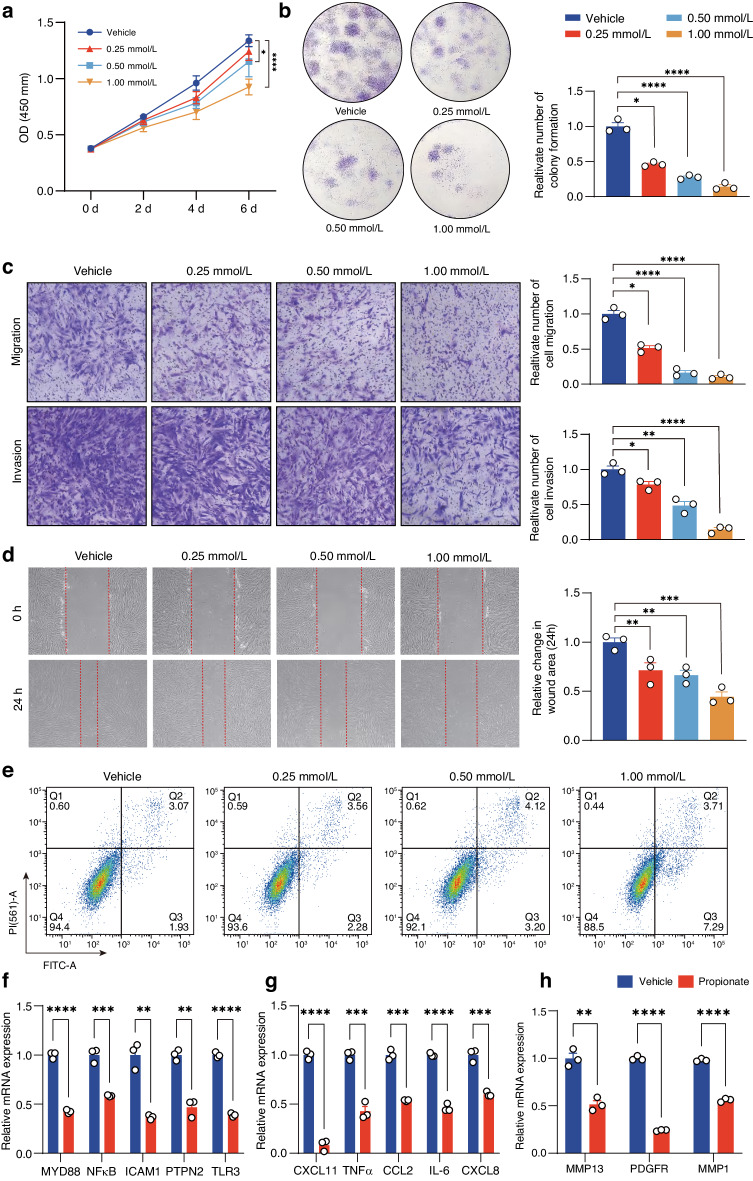
Effects of propionate on tumor-like phenotypes of RA-FLSs in vitro. **a** Viability of RA-FLSs after treatment with vehicle (PBS) or different concentrations (0.25, 0.50 and 1.00 mmol/L) of propionate for 6 days, as detected by CCK-8 assay. **b** Colony formation of RA-FLSs after treatment with vehicle (PBS) or different concentrations of propionate for 7 days. Left, colony formation images; right, quantification of the colony formation images. **c** Migration and invasion of RA-FLSs in the presence or absence of propionate for 24 h, as detected by transwell assays. Left, migration and invasion images; right, quantification of the migration and invasion images. **d** Wound healing assay for RA-FLSs in the presence or absence of propionate for 24 h. Left, wound healing images; right, quantification of the wound healing images. **e** Apoptosis of RA-FLSs after treatment with vehicle (PBS) or different concentrations of propionate, as detected by an Annexin V-FITC/PI double staining assay. Relative mRNA expression of inflammation-related genes (MYD88, NFκB, ICAM, PTPN2 and TLR3) (**f**), chemokines and inflammatory cytokines (CXCL11, TNFα, CCL2, IL-6 and CXCL8) (**g**) and migration- and invasion-related genes (MMP13, PDGFR and MMP1) (**h**) in RA-FLSs after treatment with vehicle (PBS) or propionate for 48 h. Graphs represented means ± SEM and statistical significance was calculated by two-way ANOVA (**a**), Kruskal–Wallis *H* test (**b**–**d**) and Student’s *t* test (**f**–**h**). Experiments were performed in triplicate and repeated three times. *P < 0.05, **P < 0.01, ***P < 0.001 and ****P < 0.000 1

During RA development, FLSs acquire a series of tumor-like phenotypes, including hyperproliferative capacity, enhanced migration and invasion and promoted resistance of apoptosis.^34^ In addition, RA-FLSs produce inflammatory mediators such as IL-6, IL-1β, TNF-α and MMPs.^35,36^ We examined whether propionate had effects on viability of RA-FLSs, which were characterized with positive expression of fibroblastic biomarkers THY1 and vimentin and absence of a macrophage marker CD68 (Fig. S5).^52–54^ CCK-8 and colony formation assays showed that propionate reduced proliferation of RA-FLSs in a concentration-dependent manner in vitro (Fig. 4a, b). We also examined the effects of propionate on other aggressive phenotypes of RA-FLSs. RA-FLSs treated with propionate had inhibited migration and invasion than the cells treated with vehicle, as determined by transwell assays (Fig. 4c). Wound healing assay confirmed the inhibited migration of RA-FLSs after treatment with propionate (Fig. 4d). Flow cytometric analysis demonstrated that propionate promoted apoptosis of RA-FLSs (Fig. 4e). The mRNA expression of inflammation-related genes (MYD88, NF-κB, ICAM, PTPN2 and TLR3), chemokines and inflammatory cytokines (CXCL11, TNFα, CCL2, IL-6 and CXCL8) and migration- and invasion-related genes (MMP13, PDGFR and MMP1) were lower in RA-FLSs treated with propionate when compared to those in RA-FLSs treated with vehicle (Fig. 4f–h).^55^

### Propionate disrupted HDAC3-FOXK1 interaction to reduce protein stability of FOXK1

**Fig. 5:**
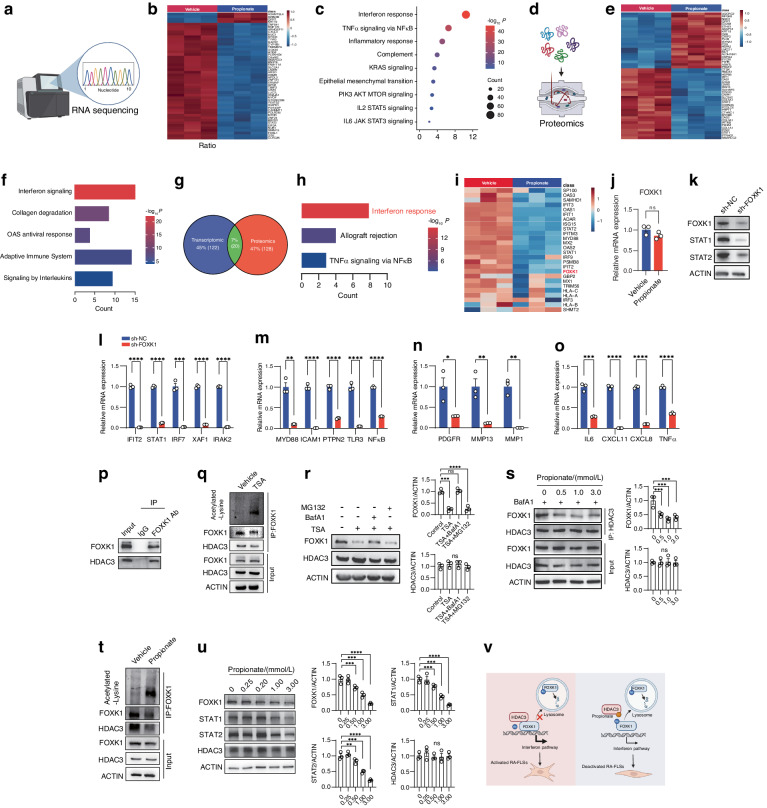
Transcriptomic and proteomic analysis of RA-FLSs after treatment with propionate. **a** Illustration of RNA sequencing of RA-FLSs after treatment with vehicle (PBS) or propionate. **b** Heatmap of differentially expressed genes in RA-FLSs after treatment with vehicle (PBS) or propionate based on RNA sequencing analysis. **c** Gene Set Enrichment Analyses (GSEA) of RNA sequencing data showing the downregulated pathways in RA-FLSs after treatment with propionate based on Hallmark Gene Sets. **d** Illustration of proteomics of RA-FLSs after treatment with vehicle (PBS) or propionate. **e** Heatmap of differentially expressed proteins in RA-FLSs after treatment with vehicle (PBS) or propionate based on proteomic analysis. **f** Functional enrichment analysis of proteomic data showing the downregulated pathways in RA-FLSs after treatment with propionate. **g** Venn diagram showing the overlap (green) of downregulated protein-coding genes between transcriptomic (blue) and proteomics (red) in RA-FLSs after treatment with propionate (*P* value < 0.05, log_2_(Flod Change) < −0.5). **h** Functional enrichment analysis of the overlapping protein-coding genes in RA-FLSs downregulated by propionate. **i** Heatmap showing differentially expressed proteins involved in interferon pathway in RA-FLSs after treatment with vehicle (PBS) or propionate. **j** Relative mRNA expression of FOXK1 in RA-FLSs after treatment with vehicle (PBS) or propionate. **k** Protein levels of FOXK1, STAT1 and STAT2 in RA-FLSs after transfection with negative control shRNA (sh-NC) or shRNA targeting FOXK1 (sh-FOXK1). Relative mRNA expression of genes involved in interferon pathway (**l**), inflammatory response genes (**m**), chemokines and inflammatory cytokines (**n**) and migration- and invasion-related genes (**o**) in RA-FLSs after knockdown of FOXK1. **p** Co-immunoprecipitation for detecting the interaction between FOXK1 and HDAC3 in RA-FLSs. **q** Level of lysine acetylation of FOXK1 in RA-FLSs after treatment with vehicle (PBS) or 5 μmol/L TSA. **r** Protein expression of FOXK1 and HDAC3 in RA-FLSs after treatment with vehicle (PBS) or 5 μmol/L TSA for 48 h, in the presence or absence of a proteasome inhibitor MG132 or a lysosome inhibitor BafA1. **s** Interaction between HDAC3 and FOXK1 in RA-FLSs after treatment with vehicle (PBS) or propionate. **t** Level of lysine acetylation of FOXK1 in RA-FLSs after treatment with vehicle (PBS) or 1 mmol/L propionate as detected by co-immunoprecipitation assay. **u** Protein expression of FOXK1, STAT1, STAT2 and HDAC3 in RA-FLSs after treatment with vehicle (PBS) or different concentrations of propionate for 48 h. **v** A schematic diagram illustrating the mechanism of action of propionate in RA-FLSs. Graphs represented means ± SEM and statistical significance was calculated by Student’s *t* test (**j** and **l**–**o**) and one-way ANOVA (**r**, **s** and **u**). Experiments were performed in triplicate and repeated three times. ns no significance, *P < 0.05, **P < 0.01, ***P < 0.001 and ****P < 0.000 1

To explore the mechanism by which propionate inhibited the pathological phenotypes of RA-FLSs, we performed RNA sequencing analysis for RA-FLSs treated with propionate or vehicle (Fig. 5a). Heatmap analysis showed that there were numerous differentially expressed genes between propionate-treated RA-FLSs and vehicle-treated RA-FLSs (Fig. 5b). Gene set enrichment analysis (GSEA) revealed that pathways involved in immunity and tumor-like transformation of RA-FLSs, such as interferon response, TNF signaling via NF-κB, inflammatory response, KRAS signaling, epithelial mesenchymal transition and interleukins (IL6 and IL2), were significantly downregulated after propionate treatment (Fig. 5c and Fig. S6). We also conducted proteomic analysis for RA-FLSs after treatment with propionate or vehicle (Fig. 5d). Differentially expressed proteins were detected between the propionate-treated RA-FLSs and the vehicle-treated RA-FLSs (Fig. 5e). Functional enrichment analysis of proteomic data demonstrated that signaling pathways including interferon response, adaptive immune system and interleukins were downregulated in propionate-treated RA-FLSs (Fig. 5f). We also performed Venn analysis to show the downregulated protein-coding genes in propionate-treated RA-FLS based on RNA sequencing and proteomic data (Fig. 5g). The overlapping protein-coding genes were mainly involved in interferon pathway (Fig. 5h), implying that propionate mainly blocked the interferon pathway to inhibit pathological phenotypes of RA-FLSs.

To explore how propionate regulates interferon pathway in RA-FLSs, we browsed all the differentially expressed proteins involved in the interferon pathway between the two groups identified by proteomic analysis (Fig. 5i). We noticed that FOXK1 was downregulated by propionate at protein level but not at mRNA level in RA-FLSs (Fig. 5i, j). Gene knockdown of FOXK1 inhibited expression of interferon pathway genes, inflammation-related genes, chemokines and inflammatory cytokines and migration- and invasion-related genes in RA-FLSs (Fig. 5k–o). Propionate is an inhibitor of the epigenetic regulator HDAC3.^40^ To test whether FOXK1 was a new substrate of HDAC3 in RA-FLSs, we performed co-immunoprecipitation assay and showed that FOXK1 interacted with HDAC3 (Fig. 5p). We manipulated HDAC3 activity using propionate or another HDACs inhibitor Trichostatin A (TSA).^56^ TSA increased acetylation of FOXK1 (Fig. 5q) and reduced protein levels of FOXK1 and key mediators of interferon signaling (STAT1 and STAT2) in RA-FLSs in a concentration-dependent manner (Fig. S7). TSA-induced degradation of FOXK1 could be reversed by a lysosomal inhibitor Bafilomycin A1 (BafA1) rather than a proteasome inhibitor MG132 (Fig. 5r), suggesting that the interaction between HDAC3 and FOXK1 is required for protecting FOXK1 from lysosomal system-mediated degradation to maintain the interferon signaling. TSA inhibited expression of interferon pathway genes (IFIT2, STAT1, IRF7, XAF1 and IRAK1), inflammation-related genes (MYD88, NF-κB, ICAM and PTPN2), chemokines and inflammatory cytokines (CXCL11, CCL2, IL-6 and CXCL8) and migration- and invasion-related genes (MMP13, PDGFR and MMP1) in RA-FLSs (Fig. S8A–D). Propionate disrupted the interaction between FOXK1 and HDAC3 in RA-FLSs (Fig. 5s). Lysine acetylation level of FOXK1 was increased in RA-FLSs after treatment with propionate (Fig. 5t). Propionate decreased protein levels of FOXK1, STAT1 and STAT2 in a concentration-dependent manner (Fig. 5u). Besides the inhibition of HDACs, SCFAs are also known to exert their functions through G protein-coupled receptor (GPCR)-induced signaling.^57^ SCFAs mostly interact with GPR41 and GPR43.^57,58^ We examined the levels of GPR41, GPR43 and HDAC3 in RA-FLSs. No expression of GPR41 and GPR43 but high level of HDAC3 was detected in RA-FLSs (Fig. S9). Based on all these results, we concluded that, under pathological condition, HDAC3 interacted with FOXK1, deacetylated FOXK1 and increased FOXK1 protein stability by inhibiting its lysosomal degradation, leading to enhanced interferon signaling and activation of RA-FLSs, while propionate disrupted HDAC3-FOXK1 interaction to reduce protein stability of FOXK1, resulting in blocked interferon signaling and deactivation of RA-FLSs (Fig. 5v).

### Propionate monotherapy or its combination with an anti-TNF drug attenuated arthritis in CIA mice

**Fig. 6:**
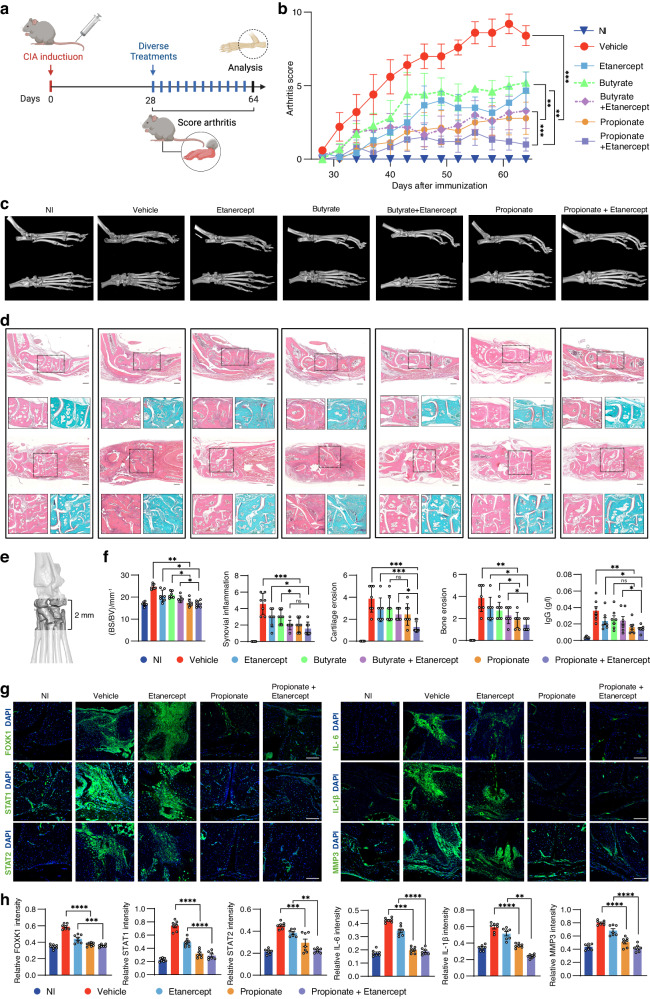
Therapeutic effects of propionate with or without the presence of an anti-TNF etanercept in CIA mice. **a** Flowchart for experimental design in CIA mice. **b** Evaluation of arthritis scores for non-immunized (NI) mice or CIA mice in different treatment groups. **c** Representative micro-CT images of the mice in different groups. **d** Images of H&E and SafO-FG staining of the paw sections. **e** Region of paws for measurement of BS/BV by micro-CT. **f** Measurement of BS/BV, and quantification of synovial inflammation and bone erosion on H&E-stained sections, as well as cartilage erosion on SafO-FG-stained sections. **g** Immunofluorescence staining of FOXK1, STAT1, STAT2, IL-6, IL-1β and MMP3 on sections of paws of the mice in different groups. **h** Quantification of fluorescence intensities of FOXK1, STAT1, STAT2, IL-6, IL-1β and MMP3 on sections of paws of the mice in different groups. *n* = 7 for each treatment group. Graphs represented means ± SEM and statistical significance was calculated by two-way ANOVA (**b**) and one-way ANOVA (**f** and **h**). ns no significance, *P < 0.05, **P < 0.01, ***P < 0.001 and ****P < 0.000 1

We investigated the potential of propionate or its combination with a biologic anti-TNF etanercept for treatment of arthritis in DBA/1J mice with CIA.^59,60^ The CIA mice were administered with vehicle, propionate, etanercept or a combination of propionate with etanercept (Fig. 6a). After the treatment, arthritic scoring showed that propionate dramatically impeded the progression of CIA when compared to vehicle (Fig. 6b). Micro-CT analysis demonstrated that the CIA mice treated with propionate showed reduced bone erosion than the mice treated with vehicle (Fig. 6c, d). Histological analysis by H&E and SafO-FG staining showed that propionate caused a notable inhibition of synovial hyperplasia, bone erosion and cartilage destruction (Fig. 6e). Measurement of BS/BV of ankle joints and quantitation of synovial score, bone erosion and cartilage erosion on H&E- and SafO-FG-stained sections consistently demonstrated the good therapeutic effects of propionate in CIA mice (Fig. 6e, f). Immunofluorescence staining demonstrated that propionate significantly decreased expression of FOXK1, STAT1, STAT2, IL-6, IL-1β and MMP3 in synovial tissues of CIA mice (Fig. 6g, h). Furthermore, we evaluated the therapeutic efficacy of the combination of propionate in the CIA mice. The combination of propionate with etanercept more significantly relieved CIA when compared to the monotherapy using etanercept (Fig. 6b–h). In addition, we examined the hepatotoxicity of propionate monotherapy and propionate in a combined therapy with etanercept. Blood biochemical assays demonstrated that the CIA mice treated with propionate or a combination of propionate with Etanercept had no obvious changes in liver function parameters including alanine aminotransferase (ALT), aspartate aminotransferase (AST), albumin (ALB) and total protein (TP) (Fig. S10).

Previously, another SCFA, i.e., butyrate, has been reported to have therapeutic potential for RA.^61^ We also tested the effects of butyrate monotherapy or its combination with an anti-TNF etanercept for treatment of arthritis in CIA mice (Fig. 6a). Arthritic scoring showed that butyrate attenuated CIA when compared to vehicle (Fig. 6b). Micro-CT analysis indicated that CIA mice treated with butyrate had reduced bone erosion (Fig. 6c). Histological analysis demonstrated that butyrate significantly inhibited synovial hyperplasia, bone erosion and cartilage destruction (Fig. 6d). The combination of butyrate with etanercept showed synergistic effects on relieving CIA when compared to the monotherapy using etanercept (Fig. 6b–f). Blood biochemical assays demonstrated that the CIA mice treated with butyrate monotherapy or its combination with etanercept had no obvious hepatotoxicity (Fig. S10). Furthermore, we compared the therapeutic effects between butyrate and propionate. Butyrate was less effective than propionate to attenuate arthritis in CIA mice after monotherapy or a combination therapy with etanercept (Fig. 6b–f).

## Discussion

Recently, the gut-joint axis hypothesis, emphasizing the interplay between host gut microbiota and joint health, has recently garnered significant attention in RA.^62^ More and more studies indicate a shift in gut microbiota composition in individuals with both preclinical and established RA, suggesting that gut microbiota may play a role in the onset and persistence of the disease. Additionally, numerous therapeutics commonly employed in managing RA have been shown to correlate with changes in the gut microbiota, hinting that manipulating these microbial communities could offer potential strategies for the prevention or treatment of RA.^63^ Nevertheless, definitive proof of a causal link between gut microbiota and RA remains elusive, and the mechanisms driving the gut-joint axis in RA are not yet fully comprehended.^64^ Deciphering the interplay between host and the gut microbial ecosystem is rather challenging.^65^ Although single-omics analyses have seen significant progress, the implementation of multi-omics strategies to synergize various layers of data from both the microbiome and the host is still at a nascent stage.^66^ In this study, we conducted a comprehensive integrative analysis of multi-omics data from both host and microbial sources to investigate the potential causal links between gut microbiota and RA, with the aim of identifying microbiome-based therapeutic strategies for RA.

Base on the newly proposed concept that genetics shape the composition of gut microbiota,^31^ we speculated that CIA-resistant C57BL/6J mouse strain and CIA-susceptible DBA/1J mouse strain might have distinct gut microbiota composition inherited from birth, which decided their predisposition to CIA.^67^ We showed that FMT between donor C57BL/6J mice and recipient DBA/1J mice conferred resistance to CIA in DBA/1J mice, implying that C57BL/6 mice might have arthritis-resistant gut microbes. We performed 16S rRNA gene sequencing using fecal samples and identified that the Bacteroides genus was more abundant in C57BL/6J mice than in DBA/1J mice. Consistently, we found that the abundance of the Bacteroides genus was enriched in HC individuals rather than in RA patients. Furthermore, earlier studies also revealed that there was low abundance of Bacteroides in new-onset untreated RA patients and immune-priming phase of CIA mice,^68,69^ suggesting that Bacteroides might be an important genus of gut microbiota associated with susceptibility to RA.

Currently, most studies focus on exploring the role of Prevotella in RA susceptibility.^68,70^ Prevotella is a genus of gut microbiota antagonistic to Bacteroides in human body.^68,70^ Relationship between different species of Prevotella and RA have been extensively characterized. For example, high abundance of *Prevotella copri* is reported to enhance RA susceptibility.^68,70^ Paradoxically, *Prevotella histicola* has shown probiotic efficacy against arthritis.^71^ However, the exact role of species of Bacteroides in RA is still not clear. We identified that *B. fragilis* was a dominant species of Bacteroides in fecal samples of C57BL/6J and the abundance of *B. fragilis* was higher in C57BL/6J mice than that in DBA/1J mice. HC individuals also had more abundant level of *B. fragilis* in fecal samples when compared to RA patients. We showed that transplantation of *B. fragilis* prevented the CIA onset and evolution in DBA/1 mice, suggesting that *B. fragilis* was a beneficial bacterial against arthritis and provided a reasonable explanation for the low susceptibility to CIA in C57BL/6J mice. This squares with the theory of the “two-hit” model for RA, with the first being some kind of microbial triggers that leads to breach of immune tolerance and a phase of clinically silent autoimmunity, and the second being an unidentified process that allows the full-blown arthritis in individuals with high susceptibility to RA.^64^ Thus, transplantation of *B. fragilis* could be potential microbe-based therapeutic option for RA.

Gut microbial metabolites have been shown to serve as a pivotal connection within the gut-joint axis in RA.^72^ Among the important class of gut microbial metabolites, SCFAs display considerable impact on host health by mechanisms related to glucose homeostasis, immunomodulation and obesity.^73^ We performed prediction of functional profiles based on 16S rRNA gene sequencing data of C57BL/6J and DBA/1J mice, and also conducted targeted and untargeted metabolomics using plasma samples of RA patients and HC individuals. Our results showed that SCFAs-related pathways, such as the TCA cycle, pyruvate metabolism, and starch and sucrose metabolism, are enriched in C57BL/6J mice and HC individuals, when compared to those in DBA/1J mice and RA patients, respectively. Moreover, level of a SCFA, i.e., propionate, was higher in C57BL/6J mice and HC individuals than that in DBA/1J mice and RA patients, respectively. This was supported by the data that C57BL/6J mice and HC individuals had higher abundance of *B. fragilis*, which produced more propionate than other SCFAs.

So far, most of the DMARDs for RA treatment are immunosuppressive drugs.^2,5^ However, besides the immune cells, structural cells of joints have also been found to participate in RA development, particularly the FLSs.^34^ RA-FLSs-directed therapies have long been suggested as a potentially alternative or complementary approach to the current immune-directed therapies in RA.^35,36,74^ In our study, we showed that propionate significantly inhibited the tumor-like phenotypes of RA-FLSs in vitro. Propionate monotherapy attenuated synovial hyperplasia, bone erosion and cartilage destruction in CIA mice. A combination of propionate with anti-TNF etanercept synergistically relieved CIA when compared to the monotherapy using etanercept. Moreover, propionate achieve better therapeutic effects in CIA mice after monotherapy or a combination therapy with etanercept than a previously identified SCFA butyrate with anti-RA effects.^61^ In addition to our findings, propionate has been documented to influence the differentiation and functions of T and B cells, thereby contributing to the maintenance of a healthy immune homeostasis and the prevention of autoimmune disorders.^75^ Thus, we speculated that propionate targeted both immune cells and structural cells in microenvironment of joints to attenuate arthritis.

HDACs are well-known to catalyze the deacetylation of various proteins, thereby affecting their stability at post-translational levels.^42^ Studies have shown that HDAC3 can interact with RCAN1 and increase RCAN1 protein stability by inhibiting its poly-ubiquitination.^76^ HDAC3 controls the stability of cyclin A by altering its acetylation level.^77^ In our study, we found that FOXK1 was another substrate of HDAC3 in RA-FLSs. Under pathological condition, HDAC3 interacted with FOXK1, deacetylated FOXK1, and increased FOXK1 stability by inhibiting its lysosomal degradation, leading to enhanced transcription of interferon signaling and activation of RA-FLSs. After treatment with propionate, propionate disrupted HDAC3-FOXK1 interaction to increase acetylation of FOXK1, resulting in reduced protein stability of FOXK1, blocked interferon signaling and deactivation of RA-FLSs. Even though propionate has also been reported to interact with GPR41 and GPR43 and activate GPCR-induced signaling,^57,58^ we detected no expression of GPR41 and GPR43 in RA-FLSs, suggesting that propionate worked on RA-FLSs in GPR41 and GPR4-independent manners. Besides, we also noticed that TNFα signaling via NFκB was also inhibited by propionate in our transcriptomics and proteomics, suggesting that TNFα signaling via NFκB might be another pathway involved in propionate-mediated deactivation of RA-FLSs.

There are also a few limitations in our study. Firstly, based on the opinion that genetics shape the gut microbiota, we examined the genetics-decided differential composition of gut microbiota between the CIA-susceptible DBA/1J and the CIA-resistant C57BL/6J mice and explored the causal relationship between gut microbiota and RA susceptibility. Even though the two genetically diverse mice strains were easy for standardized feeding and held advantage than racially different human populations to exclude the impacts of extrinsic factors, such as diet, season, smoking and infection, the exact mechanism about why genetics could decide gut microbiota in the two mouse stains is still not clear and need further investigation. Secondly, we provided new insights into the role of FOXK1 in RA-FLSs and demonstrated that FOXK1 could be a potential target for RA treatment. However, there is a lack of available FOXK1 specific inhibitors, limiting us to evaluate the therapeutic effects of FOXK1 inhibition on CIA development in vivo.

In summary, by analyzing the differential gut microbiota composition between a CIA-resistant mouse strain and a CIA-susceptible mouse strain, we revealed a causal relationship between gut microbiota and arthritis susceptibility. *B. fragilis* is identified as a beneficial gut bacterial against arthritis, which could be used for developing microbiota-oriented treatment for RA. *B. fragilis-*derived propionate inhibits HDAC3-FOXK1-interferon pathway in RA-FLSs could be used as potential therapeutics for RA.

## Materials and methods

### Cell culture

RA-FLSs were extracted from synovial tissues of RA patients and cultured in Dulbecco’s Modified Eagle’s Medium (DMEM; Corning, USA) supplemented with 20% fetal bovine serum (FBS; Corning, USA) and 1% penicillin-streptomycin (Gibco, USA). For consistency in experimental results, only RA-FLSs at passages 4–8 were utilized. The human embryonic kidney cell line 293T were obtained from American Type Culture Collection (ATCC, USA) and cultured in Dulbecco’s Modified Eagle’s Medium (DMEM; Corning, USA) supplemented with 10% FBS (Corning, USA) and 1% penicillin-streptomycin (Gibco, USA). Both cell cultures were maintained at a standard condition of 37 °C in a humidified environment containing 5% CO_2_.

### Cell viability assay

RA-FLSs were plated at a density of 2 × 10^3^ cells per well in a 96-well plate. Following an overnight incubation with 100 µL of medium to facilitate cell attachment, the cells were treated with varying concentrations of propionate (Sigma, USA) to assess proliferation over a period of 6 days. Cell viability was determined using the Cell Counting Kit-8 (CCK-8) assay. This involved washing the cells with PBS, adding 10 µL of CCK-8 reagent to each well along with 100 µL of medium, and then incubating the plates for 1 h. Absorbance was measured at 450 nm using a PerkinElmer EnSpire® spectrophotometer at two-day intervals to monitor cell viability.^78^

### Colony formation assay

RA-FLSs were plated at a density of 1 × 10^3^ cells per well into 6-well plates and allowed to adhere overnight. Subsequently, the cells were treated daily with various concentrations of propionate for a duration of 7 days. Post-treatment, the cells underwent fixation with 10% formalin for 30 min, followed by staining with crystal violet for 20 min at room temperature. High-resolution digital images of the resulting colonies were captured using a camera system. Quantitative analysis of the colony count was performed utilizing ImageJ software.

### Transwell migration assay

RA-FLSs were seeded at a density of 8 × 10^3^ cells in 100 µL of serum-free medium into the upper chamber of a Transwell polycarbonate culture insert, 6.5 mm in diameter with an 8 µm pore size (BIOFIL, USA). These inserts were placed into 24-well plates containing 600 µL of medium supplemented with 20% FBS in the lower chamber. The assay plates were incubated for 24 h with or without varying concentrations of propionate. Post-incubation, the Transwell inserts were carefully removed, and the upper chamber was cleared of non-migratory cells using a cotton swab. The cells that had migrated to the underside of the membrane were fixed with 10% formalin for 20 min, stained with 0.2% crystal violet for 30 min, and subsequently visualized under a Nikon Eclipse Ts2 microscope. To quantify the migratory cells, four distinct non-overlapping fields were counted using ImageJ software.

### Invasion assay

Matrigel Basement membrane matrix (Corning, USA) was prepared at a concentration of 300 µg/mL by dilution with PBS. This solution was then applied to the 8-µm pore size Transwell inserts. Each insert was coated with 100 µL of the diluted Matrigel and allowed to solidify at 37 °C for 30 min to form a uniform layer. Following polymerization, 8 × 10³ cells were seeded into the upper chamber of the inserts, using the same procedure as outlined for the migration assay. The assay plates were incubated for 48 h with or without varying concentrations of propionate. After the incubation period, the staining and imaging of the cells were performed using the identical protocol established for the migration assay.

### Wound healing assay

RA-FLSs were grown to confluence in six-well plates prior to performing a wound healing assay to assess cell migration. To create a wound, a sterile 200 µL pipette tip was used to scratch the cell monolayer. Following the scratch, the cells were incubated in medium with or without various concentrations of propionate. Wound images were captured at the initial time of wounding (0 h) and then again at the 24 h mark, utilizing a Nikon Eclipse Ts2 microscope equipped with CapStudio-SC200C software. The wound area was quantified using ImageJ software. The healing process was evaluated by normalizing the wound area at 24 h to that at the initial time point (0 h).

### Apoptosis assay by flow cytometry

RA-FLSs were plated in 6-well plates and exposed to varying concentrations of propionate daily for a period of three days. Post-treatment, cells were enzymatically detached and washed twice with PBS to ensure purity. Subsequently, cells were stained utilizing an Annexin V-FITC Apoptosis Detection Kit (Beyotime, CHN) to label apoptotic cells. Flow cytometric analysis was carried out using a BD flow cytometer to quantify the proportion of apoptotic cells. Data acquired from the flow cytometer were analyzed with the FlowJo software (BiotreeDB, USA) to interpret the apoptotic profiles.^79^

### Real-time PCR

RNA was extracted employing the RNeasy kit (TransGen Biotech, China) following the manufacturer’s protocol. cDNA was synthesized using the PrimeScriptTM RT reagent Kit with gDNA Eraser Kit (TaKaRa, JAN). The primers were synthesized by Sangon Biotech Co., Ltd and listed in Table S1. For real-time PCR, a 20 µL volume of the final PCR solution was prepared by adding 5 µL of diluted cDNA product, 10 µL of 2 × Power SYBR® Green PCR Master Mix (TransGen Biotech, CHN), and 5 µL of each forward and reverse diluted primer. The amplification and detection were conducted on the Bio-Rad CFX 96 Touch System, with all samples run in technical triplicates to ensure data reliability. Quantitative data were generated and analyzed using the Bio-Rad CFX Manager software.

### RNA sequencing and data analysis

RA-FLSs were seeded in 6-well plates. Following overnight incubation, the cells were treated with 1 mmol/L propionate or vehicle for 48 h. Total RNA samples were obtained by adding 1 mL of Transzol (Transgen, USA) for subsequent transcriptome sequencing by BGI Genomics. Briefly, sequencing libraries were generated after depleting ribosomal RNA, synthesizing cDNA, and ligating adapters using DNBSEQ Eukaryotic mRNA library (BGI Genomics, CHN). After cluster generation, the libraries were sequenced using the DNBseq platform (BGI Genomics, CHN) and 150 bp paired-end reads were generated. Raw data with adapter sequences or low-quality sequences were filtered using the SOAPnuke software (BGI Genomics, CHN). The resulting data was then converted and stored in the fastq format for subsequent analysis. To assess the quality of the data, FastQC version 0.11.9 was utilized to generate a quality control report. The raw sequencing reads were then aligned to the reference genome GRCh38 using HISAT2 version 2.2.1 and the aligned reads were sorted by coordinate using Samtools version 1.10. The gene expression counts were quantified using FeatureCounts version 1.10, and gene expression counts were normalized for differential analysis using DESeq2 version 4.2.2, with an FDR threshold of 0.05.

### Sample preparation for proteomics

Protein extracts from RA-FLSs were prepared using the EasyPep Mini MS Sample Prep Kit (Thermo Fisher Scientific, USA).^80^ Initially, cells were lysed with the provided lysis buffer to extract proteins. A quantity of 100 µg of the protein extract was then transferred to a fresh microcentrifuge tube, and the volume was brought up to 100 µL using the same lysis buffer. Subsequent steps involved the addition of reduction and alkylation solutions to the protein sample. The mixture was gently mixed and subjected to incubation at 95 °C for 10 min to facilitate the reduction and alkylation processes. Post incubation, the sample was allowed to cool to room temperature. The reconstituted Trypsin/Lys-C Protease Mix was then introduced to the prepared protein sample, followed by incubation with shaking at 37 °C for 2 h, to achieve protein digestion. Upon completion of the digestion, the digestion stop solution was added to terminate the enzymatic reaction. Peptides were then purified using a peptide clean-up column. The samples were dried via vacuum centrifugation and subsequently reconstituted in a 0.1% formic acid aqueous solution, preparing them for LC-MS/MS analysis.

### Nanoflow LC-MS/MS analysis

The Orbitrap Fusion mass spectrometer (Thermo Fisher Scientific, USA) was used in combination with an Easy-nLC 1000 ultrahigh-pressure liquid chromatography pump (Thermo Fisher Scientific, USA) for the LC-MS/MS analysis. Separation was achieved using a trap column and an analytic column with a spray tip, both filled with 3 µm/120 Å ReproSil-Pur C18 resins (Dr. Maisch GmbH, DE). The separation buffers were comprised of 0.1% formic acid in both water and acetonitrile. A fraction of the collected samples was initially introduced into the trap column with a 2 µL/min flow rate, and then, it was separated via the analytical column at a flow rate of 300 nL/min. The separation gradient was established as starting with 3%–7% acetonitrile over 2 min, increasing to 22% acetonitrile over the next 50 min, then to 35% acetonitrile in 10 min, surging to 90% acetonitrile within 2 min, maintaining at 90% for 6 min, dropping back to 3% acetonitrile in 2 min, and finally stabilizing at 3% acetonitrile for a duration of 13 min. Full MS scans were performed in an Orbitrap mass analyzer over m/z range of 395–1 205 with a mass resolution of 60 000. Data was processed and analyzed for DIA-Based proteomics using Spectronaut version 14.9 (Biognosys, CH).^81^

### Plasmid constructs, lentivirus packaging and infection

pLKO.1-U6-EF1a-copGFP-T2A-puro were purchased from IGE BIO (CHN). Plasmid constructs were created in accordance with the protocol available on the Addgene website. The FOXK1 shRNA sequence utilized was as follows: CCATCAAGATCCAGTTCACGT. The shRNA sequence was synthesized by Sangon Biotech (CHN), and subsequently, it was digested with a restriction enzyme and ligated into the vector. All plasmids were confirmed by DNA sequencing. For lentivirus packaging and infection, HEK293T cells were seeded onto a 6 cm dish in a quantity of 6 × 10^5^. The co-transfection procedure was conducted after a duration of twenty-four hours, utilizing 1 mg of target plasmids, 0.75 mg of psPAX2, and 0.25 mg of pMD2.G. The media of the culture was replaced six h following the transfection process. The initial batch of material was collected 48 h after transfection, without any further changes to the medium. After a period of medium refreshment, a further collection of media took place 72 h after the transfection. The two medium batches were combined and subjected to centrifugation at 1 250 r/min for five min to remove any cell debris. The resulting supernatant was then kept in 1 mL portions at a temperature of −80 °C. In the same manner, 6 × 10^5^ RA-FLSs were placed in a 6 cm plate and, after 24 h, were given 1 mL of the thawed lentivirus solution, which was gently stirred to ensure thorough mixing. The medium was substituted 24 h subsequent to the introduction of the virus. The process of antibiotic selection was began 48 h after transfection, using a concentration of 2 mg/mL puromycin in the media. This selection continued until the majority of cells died, leaving only the resistant cells, which finally attained confluency. Following that, the cells were prepped for extended preservation in liquid nitrogen.

### Western blotting

Western blot analysis was conducted as previously described.^79^ In brief, total protein levels were quantified and loaded onto an SDS-PAGE gel. The separated proteins were then transferred onto a PVDF membrane (Millipore, MA, USA) via the Bio-Rad Trans-Blot Turbo™ transfer apparatus (USA). Following blocking with 5% non-fat dry milk in TBST, the membrane was incubated overnight at 4 °C with primary antibodies against FOXK1 (1:1 000, Abclonal, CHN), STAT1 (1:1 000, proteintech, CHN), STAT2 (1:1 000, proteintech, CHN), HDAC3 (1:1 000, proteintech, CHN) and β-actin (1:2 000, Abclonal, CHN). Following this, the membrane was rinsed thrice using TBS-T and then incubated with suitable HRP-linked secondary antibodies for one h at room temperature. Chemiluminescent detection was subsequently carried out using the enhanced chemiluminescence kit (ABclonal, CHN) and the blots were visualized using the Tanon Multi5200 chemiluminescence imaging system (Tanon, Multi5200, CHN).

### Immunoprecipitation and ubiquitination assay

The immunoprecipitation procedure was performed as previously described.^82^ 5 × 10^4^ RA-FLSs were incubated with propionate for 24 h and were lysed in lysis buffer (Thermo Scientific, USA) with a proteinase inhibitor cocktail. The lysate was centrifuged at 13 000 × *g* at 4 °C, and the resulting supernatant was incubated overnight at 4 °C with anti-FOXK1 primary antibody (1:100, Abclonal, CHN) or anti-HDAC3 (1:100, Abclonal, CHN). Subsequently, the mixture was attached to Protein A/G Magnetic Beads (Thermo Scientific, USA) at room temperature for 1 h. The beads are then washed five times extensively to remove non-specifically bound proteins by using DynaMag™-2 Magnet (Invitrogen, USA). The immunoprecipitated proteins were then prepared in loading buffer, heated at 100 °C for 10 min, and subjected to SDS-PAGE and western blot analysis using primary antibodies against acetylated-Lysine (1:1 000, PTM BIO, CHN), FOXK1 (1:1 000, Abclonal, CHN), HDAC3 (1:1 000, proteintech, CHN) and β-actin (1:2 000, Abclonal, CHN).

### Mice

Male C57BL/6J and DBA/1J mice aged 6-8 weeks were obtained from GemPharmatech (CHN). All animal studies were performed at the Experimental Animal Center of the Southern University of Science and Technology. This facility guarantees a controlled environment free from pathogens, offers unrestricted access to food and water, maintains a consistent temperature of 22 °C, and follows a 12-h light/dark cycle. The study’s methods were approved by the Institutional Animal Care and Use Committees (IACUC) at Southern University of Science and Technology, guaranteeing adherence to recognized animal care criteria.

### CIA mouse model

The CIA model was performed as previously described.^83^ In brief, an emulsion was formed by combining bovine type II collagen (Chondrex, USA) with an equal amount of Complete Freund’s Adjuvant (Chondrex, USA). The mice were administered a single subcutaneous injection near the base of their tail. The injection consisted of 100 µL of an emulsion containing 100 µg of collagen and 2 mg/mL of Mycobacterium tuberculosis. Two independent observers, who were unaware of the therapy groups, did the assessment of arthritis severity using clinical arthritic scoring. The scoring system encompassed a range of values from 0 to 4, where 0 denoted the absence of symptoms, 1, indicated the presence of redness and/or swelling in one joint, 2, indicated the presence of redness and/or swelling in more than one joint, 3, revealed redness and/or swelling over the entire paw, and 4, indicated the presence of severe deformity and/or ankylosis. The scoring of each paw of the mouse resulted in a maximum achievable score of 16 per mouse. Mice with a score of one or above were categorized as exhibiting arthritis.

### FMT experiment

Fecal samples were obtained from both C57BL/6J and DBA/1J mice. These samples, immediately after collection, were suspended in a saline solution at a concentration of 30 mg of feces/mL saline, then thoroughly homogenized using a TissueLyzer (Powteq, CHN), and subsequently passed through a stainless-steel mesh with 25 µm openings. Following this, the fecal solutions were combined with 10% sterile glycerol (Beyotime, CHN), portioned into aliquots, and preserved at −80 °C for future use. DBA/1J mice received an oral dose of 200 µL of this fecal concoction twice weekly until the study concluded.

### Bacteria strain administration

*B. fragilis* (ATCC #23745, USA) were cultured as per the guidelines provided by the manufacturer. DBA/1J mice were subjected to an antibiotic regimen via oral gavage, receiving 1 g/L of a combination of ampicillin, neomycin, and metronidazole. A week following this, the mice were administered the specified bacterial strain through gavage twice weekly for the duration of the study, with anaerobe basal broth (Solarbio, CHN) serving as the vehicle control. Furthermore, the functional profiles of the microbiota in C57BL/6J and DBA1/J mice were inferred from 16S rRNA gene sequencing data, utilizing the Phylogenetic Investigation of Communities by Reconstruction of Unobserved States (PICRUSt2) software in conjunction with the MetaCyc database for analysis.^84^

### Fecal DNA extraction, 16S rRNA gene sequencing, and data analysis

Microbial DNA extraction and 16S rRNA amplicon sequencing were performed by MAGIGENE. Following their preparation, the amplicon libraries were sequenced on the MiSeq system (Illumina, USA). The sequences obtained from the bacteria were then organized into Operational Taxonomic Units (OTUs) and matched to the Greengenes microbial gene database, ensuring a 97% sequence similarity threshold, employing the QIIME2 software (version 2022.11).^85^ OTUs present in fewer than 10 samples were removed. The final Biological Observation Matrix files were derived from mouse samples, with an average count of 81 255 per sample, and were used for further analyses. The summarization of bacterial taxonomy, assessment of microbial diversity through rarefaction analysis, and evaluation of compositional variations were performed using the designated script provided by QIIME2.^85^ PCA plots and a heatmap were generated based on the normalized bacterial abundance in R.

### Collection of human samples

Plasma samples from RA patients and healthy control (HC) subjects were obtained from the Department of Laboratory Medicine at Peking University Shenzhen Hospital and the Department of Rheumatology at Guanghua Hospital, affiliated with Shanghai University of Traditional Chinese Medicine. The procedures for collecting and processing these samples were consistent across all participants. The demographic and clinical characteristics of the RA patients and HC subjects are detailed in Table S2. Prior to participation, informed consent was secured from all individuals involved in the study, which received ethical approval from the Clinical Ethics Committees at both Peking University Shenzhen Hospital and Guanghua Hospital of Shanghai University of Traditional Chinese Medicine.

### Targeted metabolomics data acquisition and processing

Fecal and plasma SCFA contents were quantified following the established protocol with some modifications.^86^ Fecal samples were processed by extracting about 150 mg portions using a TissueLyzer with a 0.005 mol/L NaOH solution, which included 1 µg/mL of 2-methylbutyric acid as an internal standard (IS). After centrifuging these extracts at 13 200 r/min for 15 min, the supernatants were moved to 10-mL glass tubes. Plasma samples were prepared by combining 300 µL of the sample with 500 µL of a 0.005 mol/L NaOH solution containing the IS, mixed in a glass centrifuge tube. This was followed by the addition of 500 µL of a propanol/pyridine (PrOH/Py) solvent mix and 100 µL of perchloric acid (PCF), vortexed and sonicated for 1 min. The propyl derivatives extracted with hexane were then ready for GC-MS analysis. The analysis was performed on an Agilent Technologies GC/MS system, injecting 1 microliter of extract in a split mode (1:6), with separation on an Agilent HP-5ms column and helium as the carrier gas. The oven temperature began at 50 °C, increasing through a series of ramps to a final 290 °C, which was maintained for 8 min. The method’s precision, both intra- and inter-day, was maintained below 15%, and compound identification utilized the NIST 14.0 database.

### Untargeted metabolomics data acquisition and processing

To prepare plasma samples for analysis, 100 µL of plasma was combined with 400 µL of a 1:1 acetonitrile:methanol extraction solution containing a mix of isotopically-labeled internal standards. The mixture was vortexed for 30 s, sonicated for 10 min in ice-water, and then incubated at −40 °C for 1 h. Post-centrifugation at 12 000 r/min for 15 min at 4 °C, the supernatant was transferred to a fresh glass vial for further analysis. A quality control (QC) sample was created by pooling equal volumes of supernatant from all samples. LC-MS/MS analysis utilized a Vanquish UHPLC system connected to a Q Exactive HFX Orbitrap mass spectrometer, with separations on a UPLC BEH Amide column. The mobile phase comprised 25 mmol/L ammonium acetate and ammonia hydroxide in water (pH 9.75) and acetonitrile. With a 2 µL injection volume and a 4 °C auto-sampler temperature, the system acquired data in information-dependent acquisition (IDA) mode via Xcalibur software. ESI source settings included sheath and auxiliary gas flows, capillary temperature, full MS and MS/MS resolutions, collision energy levels, and spray voltage for both positive and negative modes. Data conversion to mzXML was done using ProteoWizard, followed by peak processing with XCMS. Metabolite identification employed an in-house database with a 0.3 cutoff. MetaboAnalyst 5.0 facilitated metabolite enrichment and visualization through PCA, Orthogonal PLS-DA, and heatmaps. MetaMapp was used for metabolomic network analysis, visualized in Cytoscape 3.9.1.^87^

### In vivo administration of SCFAs and etanercept

28 days following the initiation of arthritis, DBA/1J mice received treatments that included either 200 mmol/L sodium butyrate or 200 mmol/L sodium propionate (both from Sigma, USA) added to their drinking water, and/or were given intraperitoneal injections of 5 mg/kg etanercept (sourced from MCE, USA) twice every week until the conclusion of the study.

### Histological analysis

Mouse hind paws were dissected, fixed overnight in 10% formalin at 4 °C, and subsequently decalcified in 10% EDTA solution at 4 °C over a period of three weeks. The tissues were then processed, embedded, and sectioned at a 5 µm thickness for H&E and Safranin O-Fast Green staining. Histopathological evaluation was conducted blindly, following previously established protocols.^88^ Images of the entire ankles were acquired using a NanoZoomer S60 Digital slide scanner C13210-01 (HAMAMATSU, JAN) and analyzed using NDP.view2 software (HAMAMATSU, JAN).^88^

### Micro-CT analysis

Mouse ankle specimens were immersed in 10% formalin for fixation and subsequently placed in 70% ethanol in preparation for imaging. High-resolution micro-computed tomography (µCT) scanning was conducted using a Bruker Skyscan 1276 scanner in the USA, capturing images at a 10 µm resolution under settings of 60 kV/100 mA through a 0.5 mm aluminum filter. The scanned data were then reconstructed into three-dimensional representations with the aid of NRecon and DataViewer software (Bruker, USA). Further data analysis was executed using CTAn software, while CTvox software was employed to produce the 3D visualizations, all of which are products of Bruker, USA.

### Immunofluorescence staining

To perform immunofluorescence staining on tissue sections, 5 µm thick slices were subjected to deparaffinization using xylene and progressively hydrated with gradient of ethanol to water. Then, the sections were subjected to antigen retrieval, permeabilized with 0.2% Triton X-100. Blocking was carried out with 5% BSA for 1 h before incubating overnight at 4°C with primary antibodies against FOXK1 (1:100), STAT1 (1:100), STAT2 (1:100), IL-6 (1:100, Abcam, UK), IL-1β (1:100, Abcam, UK) and MMP3 (1:100, Abcam, UK). The sections were then rinsed and incubated with either anti-mouse or anti-rabbit Alexa Fluor 488 secondary antibodies (1:200, Abcam, UK) for 1 h at room temperature. Finally, the sections were counterstained with DAPI (Beyotime, CHN) and images were acquired using a confocal fluorescence microscope (Leica, SP8, DE).

### Serum biochemical assays

Blood samples were collected from mice after treatment via hepatic portal vein puncture. Serum was collected via centrifugation of blood samples in 12 000 × *g* at 4 °C for 30 min. Serum biochemical parameters including alanine aminotransferase (ALT), aspartate aminotransferase (AST), albumin (ALB), total protein (TP), and lgG were analyzed by a MS-480 Automatic Biochemistry Analyzer (Medicalsystem Biotechnology, CHN).

### Statistical analysis

Data analysis was conducted using GraphPad Prism software. For assessing differences among multiple independent groups, one-way ANOVA followed by a post-hoc test was employed. Comparisons between two independent groups were made using the Student’s *t* test. For examining differences among groups categorized by two factors, two-way ANOVA with a subsequent post-hoc test was utilized. Data in the figures are represented as mean ± SEM, with levels of statistical significance denoted by *P < 0.05, **P < 0.01, ***P < 0.001, and ****P < 0.000 1. Sample sizes for in vivo studies were established based on power analysis beforehand. Mice were assigned to groups in a random and blinded manner, and any mice in poor health prior to the start of the studies were not included.

### Supplementary information

Supplementary information
