# Supplementary material for: Gut microbial metabolite targets HDAC3-FOXK1-interferon axis in fibroblast-like synoviocytes to ameliorate rheumatoid arthritis
Source: Bone Res. 2024 May 23;12:31. doi: 10.1038/s41413-024-00336-6 (PMC11116389; doi:10.1038/s41413-024-00336-6)
Supplement: Supplementary file 1 — Supplementary information [file 41413_2024_336_MOESM1_ESM.docx]

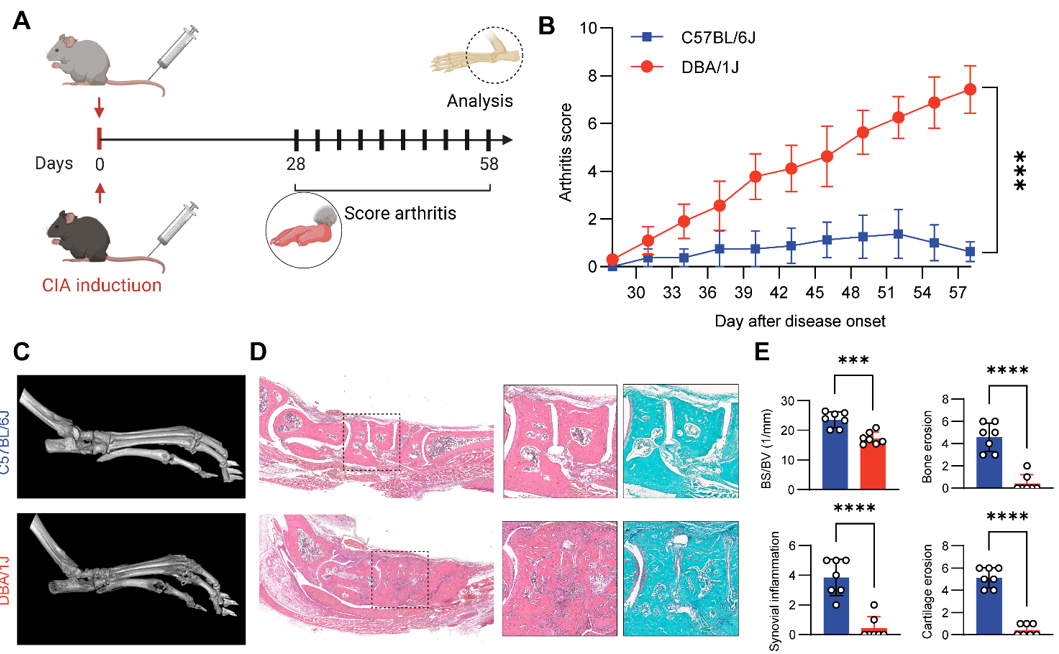


**Fig. S1.** **Establishment of CIA in** **DBA1/J mice and C57BL/6J mice.** **(A)** Illustration of CIA establishment. Briefly, CIA is induced in DBA1/J mice or C57BL/6J mice by intradermal injection of an emulsion consisting of collagen II and an adjuvant into the tail base. **(B)** Evaluation of arthritis scores. **(C)** Representative micro-CT images of the mice. **(D)** Images of H&E and SafO-FG staining of the paw sections. **(E)** Measurement of BS/BV, and quantification of synovial inflammation and bone erosion on H&E-stained sections, as well as cartilage erosion on SafO-FG-stained sections. n = 7 for each treatment group. Graphs represented means ± SEM and statistical significance was calculated by two-way ANOVA (B) and Student’s *t*-test (E). **P* < 0.05, ***P* < 0.01, ****P* < 0.001 and *****P* < 0.0001.


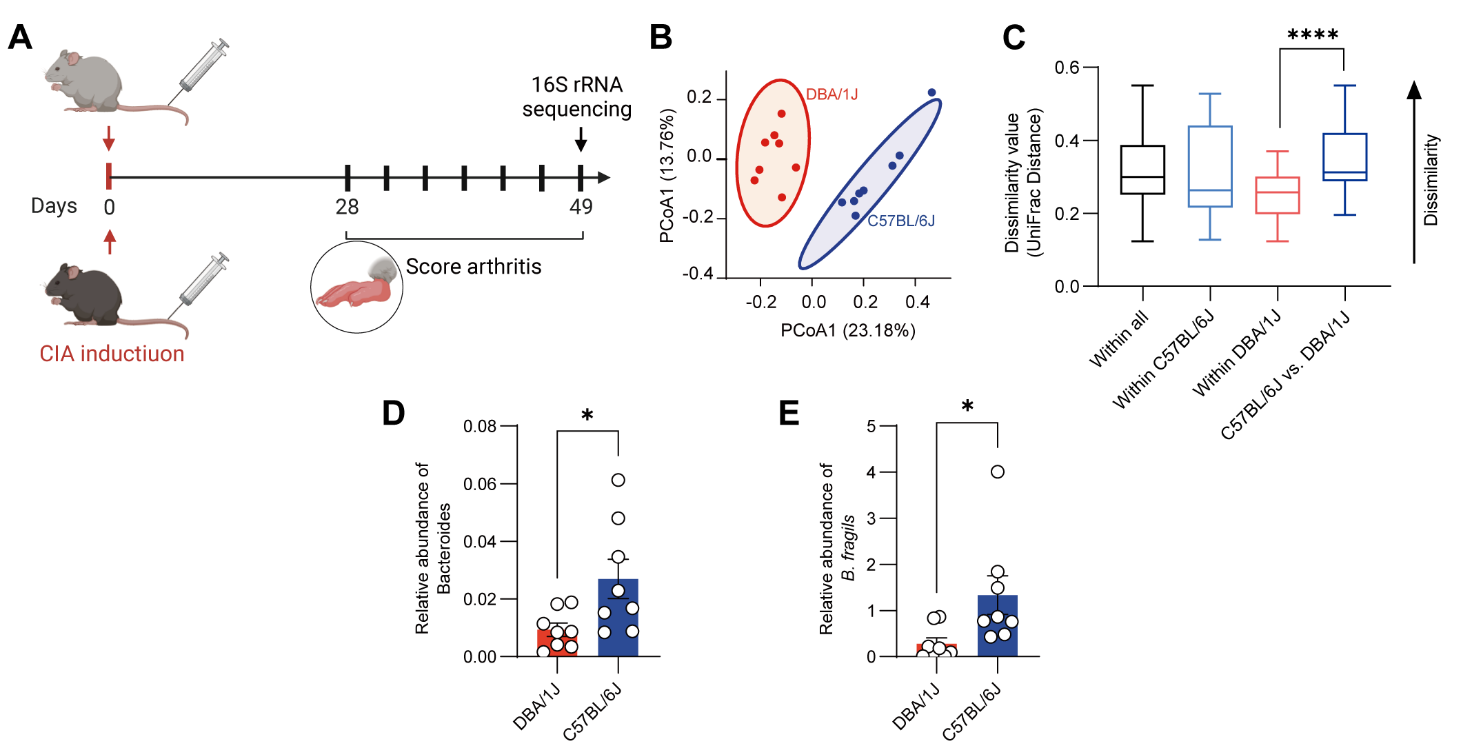


**Fig. S2. Differential composition of gut microbiota between C57BL/6J mice and DBA/1J mice** **with established CIA. (A)** Illustration of the 16S rRNA sequencing using fecal bacterial DNA isolated from DBA/1J and C57BL/6J mice with established CIA. **(B)** PCoA plots of 16S rRNA gene sequencing data depicting the differential microbial composition between C57BL/6J mice and DBA/1J mice with established CIA. **(C)** Boxplots showing unweighted UniFrac distances within and between samples. **(D)** Relative abundance of Bacteroides in feces of C57BL/6 and DBA/1J mice with established CIA as detected by 16S rRNA sequencing. **(E)** Relative abundance of *B. fragilis* in feces of C57BL/6 and DBA/1J mice with established CIA as detected by real-time PCR. n = 8 for each treatment group. Graphs represented means ± SEM and statistical significance was calculated by one-way ANOVA (C) and Student’s *t*-test (D and E). **P* < 0.05, ***P* < 0.01, ****P* < 0.001 and *****P* < 0.0001.

­­
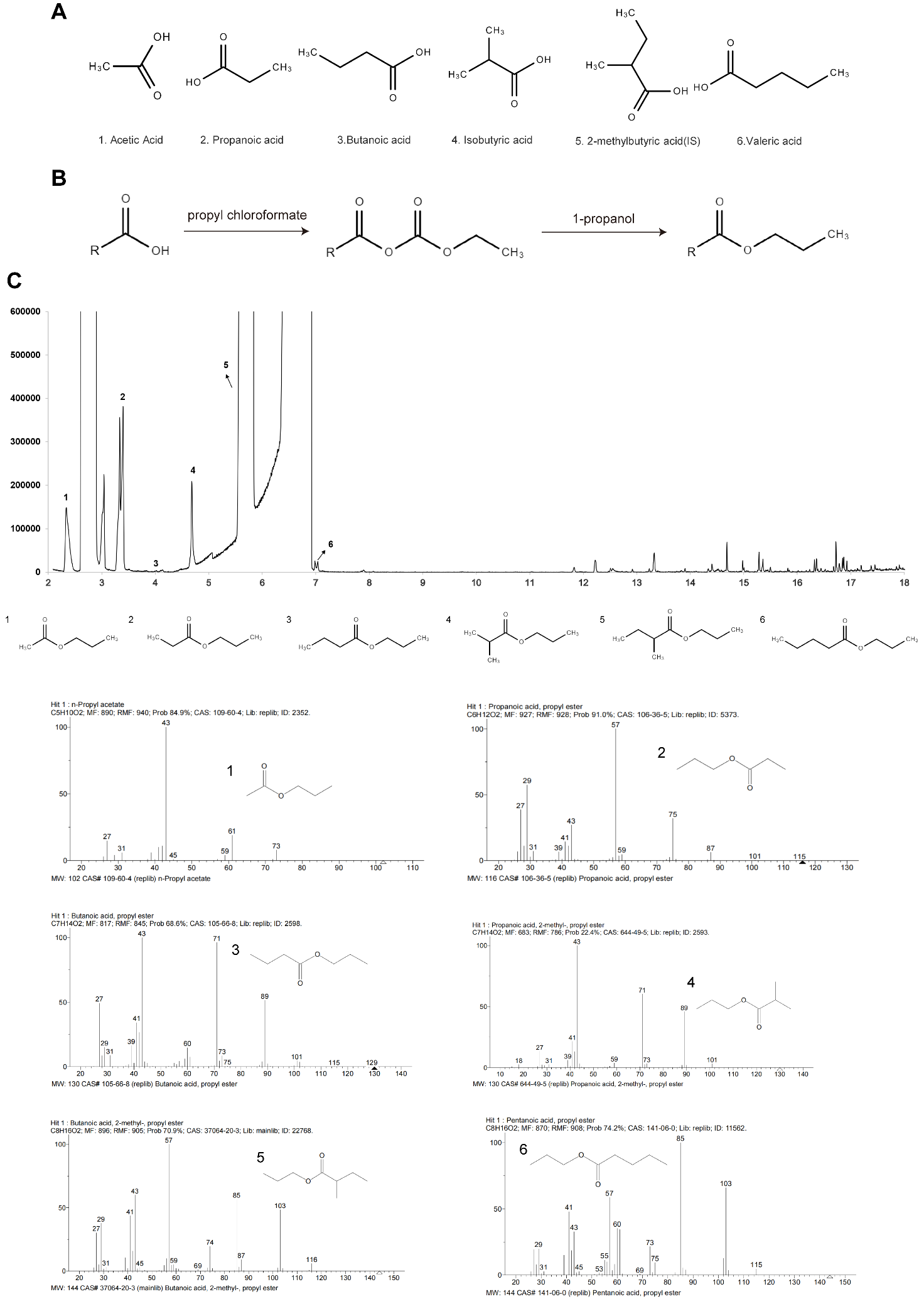


**Fig. S3. Targeted metabolomics of SCFAs after propyl chloroformate derivatization. (A)** Structure of 5 SCFAs and Internal s­tandard (IS) detected by GC-MS. **(B)** Reaction scheme showing the derivatization of SCFAs using propyl chloroformate (PCF). The R represents an alkyl group. (**C)** Total ion chromatogram SCFAs. **(D)** Structure determination report of SCFAs.


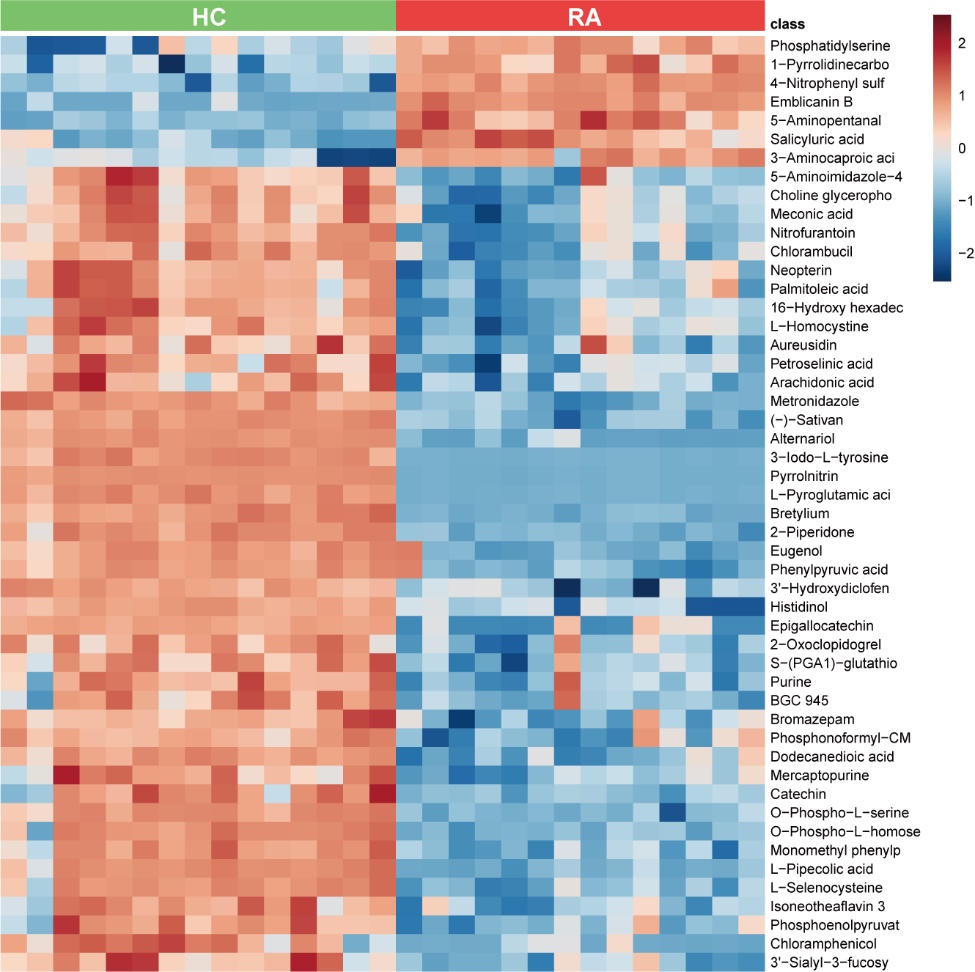


**Fig. S4. Metabolic alterations between HC individuals and RA patients by untargeted metabolomics.** Hierarchical clustering heatmap representing top 50 significantly changed metabolites between HC individuals and RA patients.


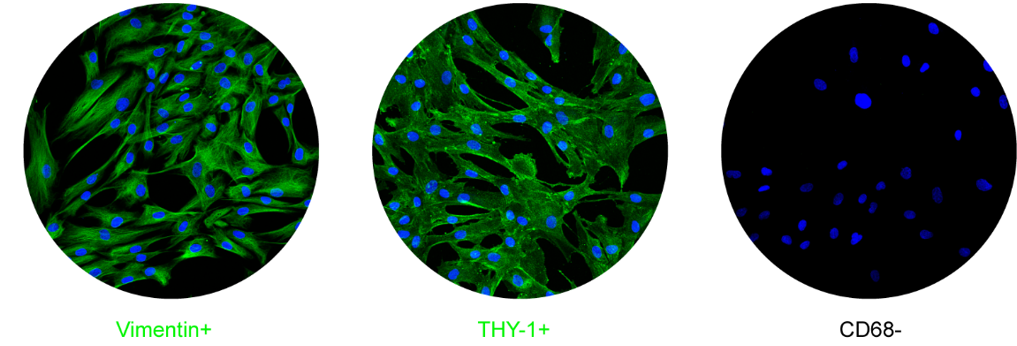


**Fig. S5. Characterization of RA-FLSs.** Immunofluorescence staining for fibroblastic biomarkers (vimentin and THY-1, green) and a macrophage marker CD68 (green) in RA-FLSs. Cell nuclei were counterstained with DAPI (blue). Experiments were performed in triplicate and repeated for three times.


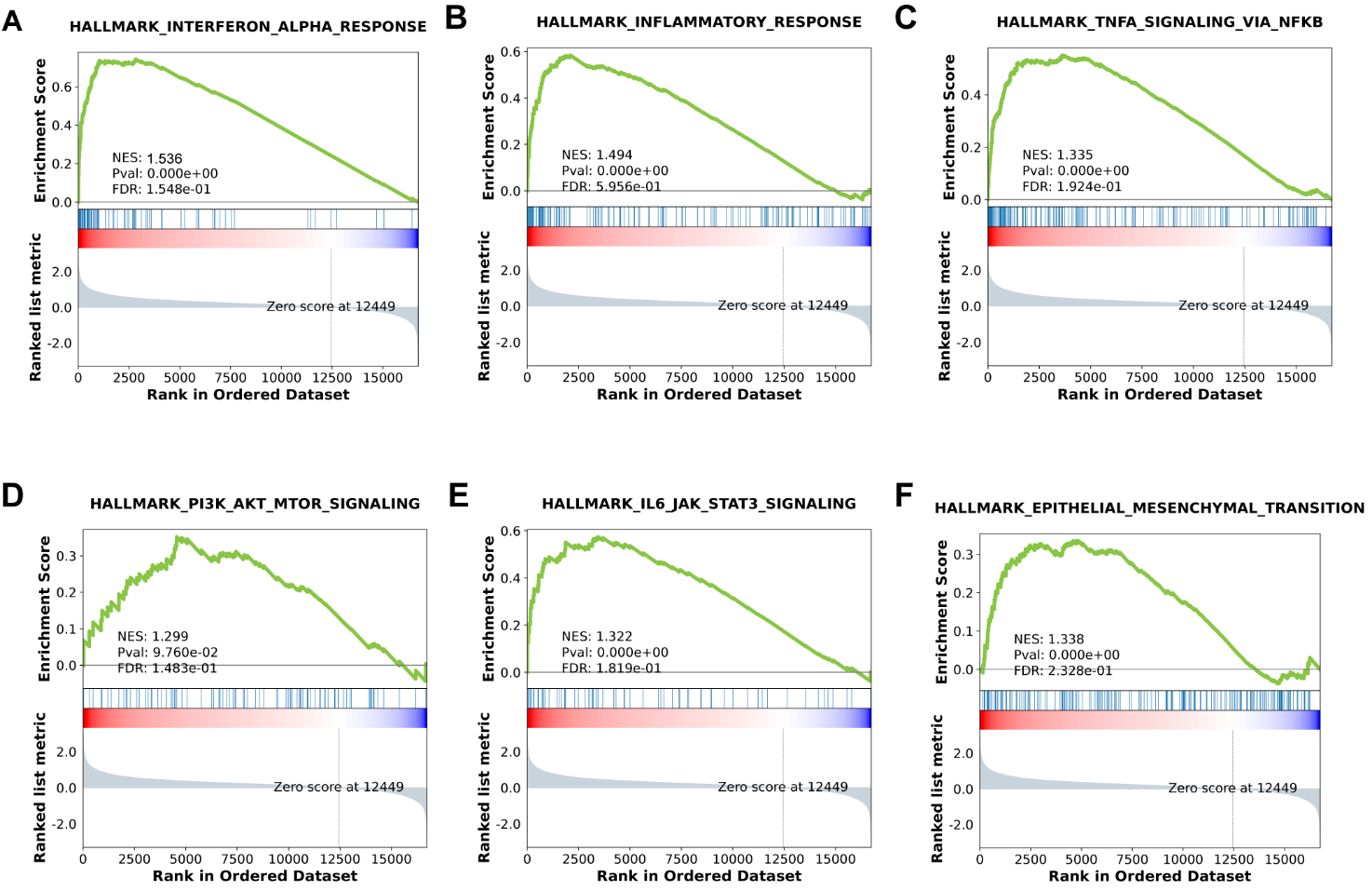


**Fig. S6. Transcriptomic analysis _­­­­_of RA-FLSs after treatment with propionate. (A-F)** GSEA of the interferon alpha response **(A)**, inflammatory response **(B)**, TNFα signaling via NF-κB **(C)**, PIK3-AKT-MTOR signaling **(D)**, IL-6-JAK-STAT3 signaling **(E)**, and epithelial-mesenchymal transition (EMT) **(F)** in propionate-treated RA-FLSs. Experiments were performed in triplicate and repeated for three times.


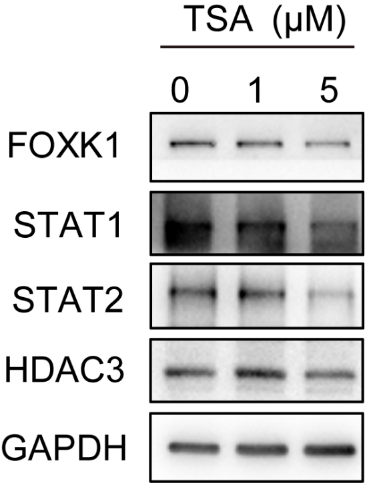


**Fig. S7. Effects of TSA on** **levels of FOXK1, STAT1, STAT2 and HDAC3 in RA-FLSs *in vitro*.** Protein levels of FOXK1, STAT1, STAT2 and HDAC3 in RA-FLSs after treatment with vehicle (PBS) or different concentrations of TSA. Experiments were performed in triplicate and repeated three times.

­­­­
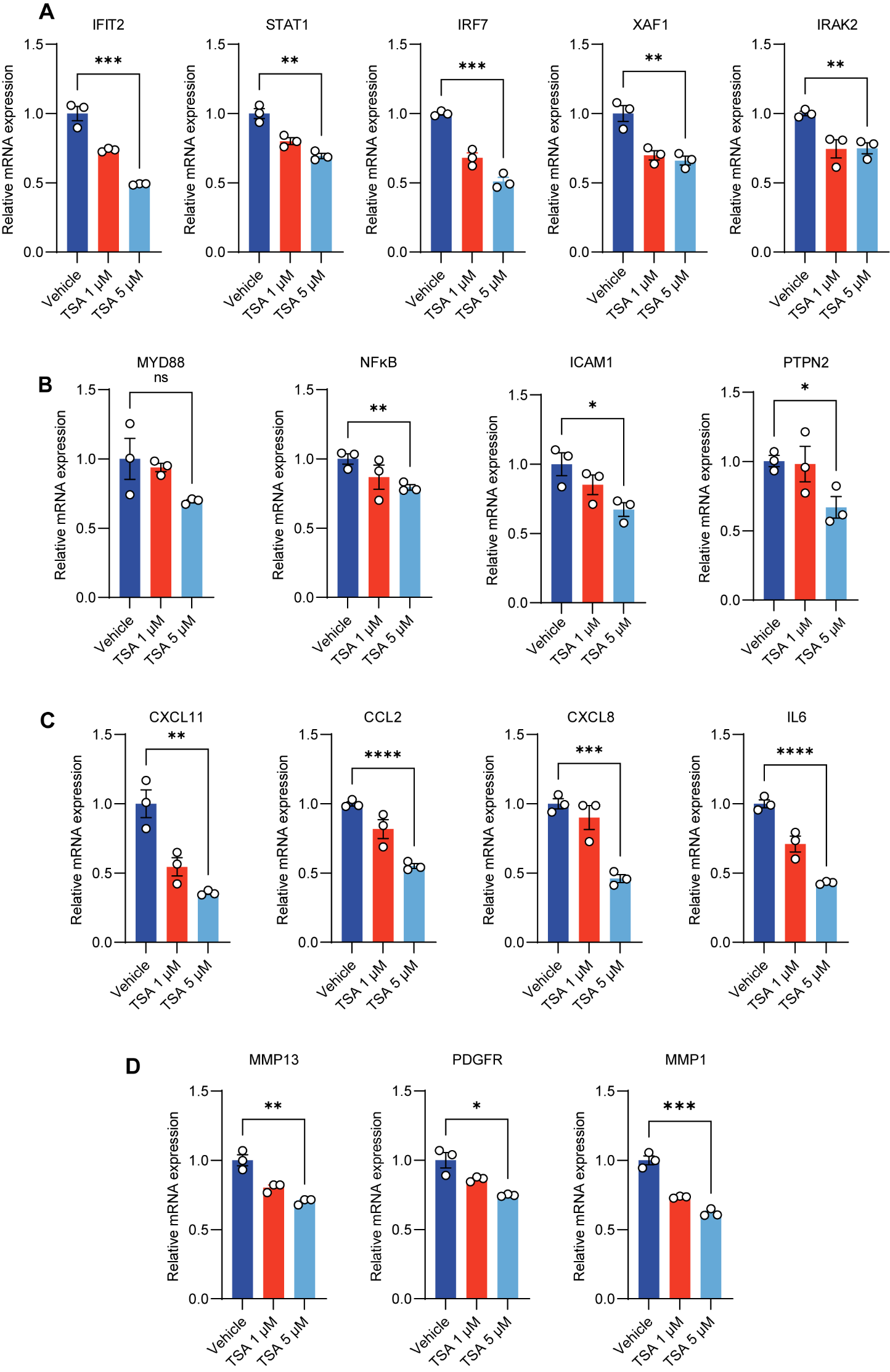


**Fig. S8. Effects of TSA on pathological phenotypes of RA-FLSs *in vitro*. (A-D)** Expression of genes involved in interferon pathway **(A),** inflammatory response genes **(B)**, chemokines and inflammatory cytokines **(C)** and migration- and invasion-related genes **(D)** in RA-FLSs after treatment with vehicle (PBS) or TSA. Graphs represented means ± SEM and statistical significance was calculated by one-way ANOVA. Experiments were performed in triplicate and repeated three times. **P* < 0.05, ***P* < 0.01 ****P* < 0.001 and *****P* < 0.0001.


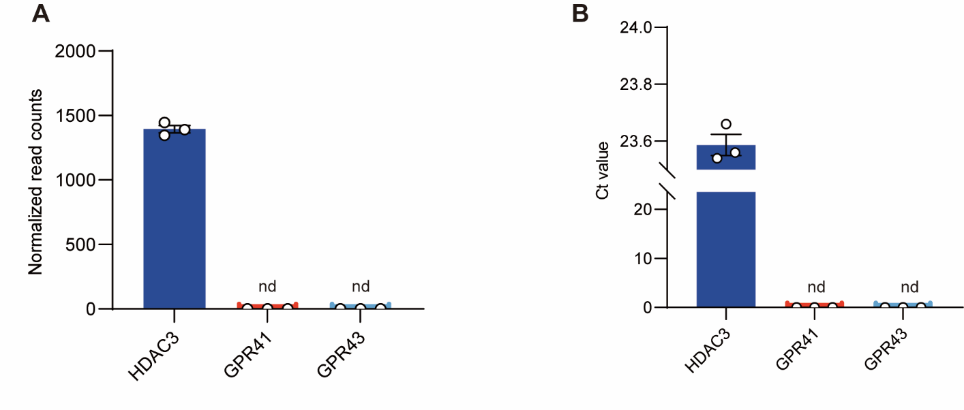


**Fig. S9. The expression of GPRs and HDAC3 in RA-FLSs.** The Ct values of GPR41, GPR43 and HDAC3 were detected by real-time PCR. nd, not detected. Experiments were performed in triplicate and repeated three times.


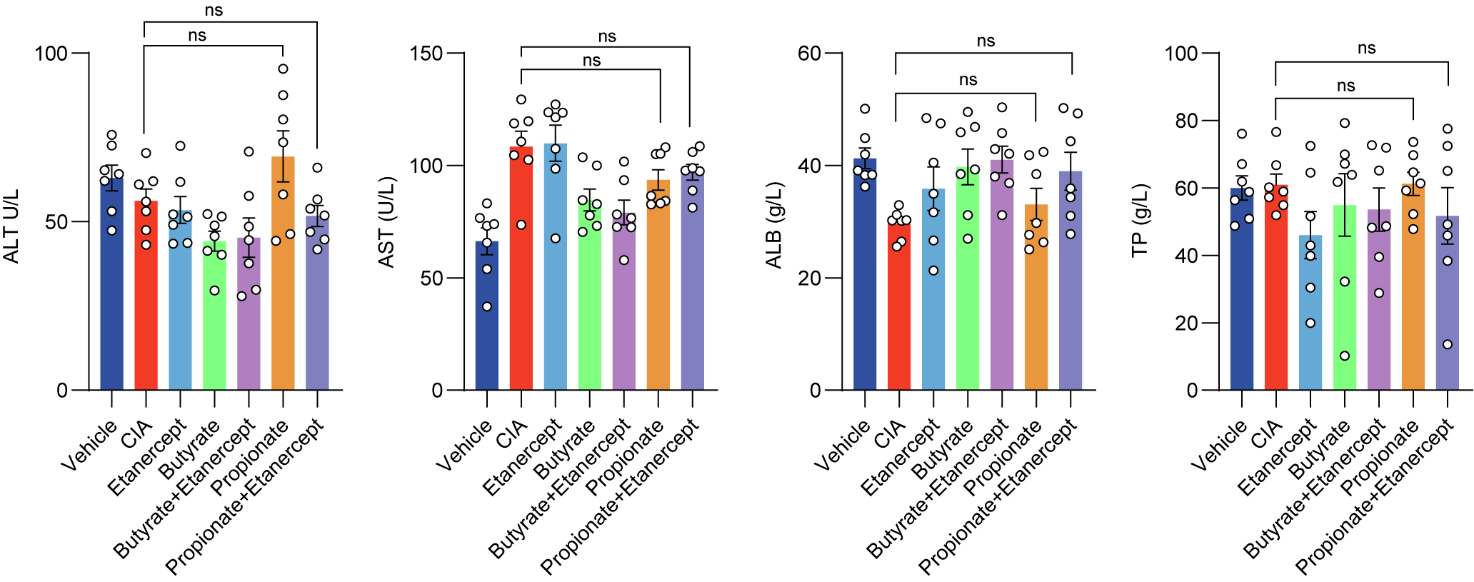


**Fig. S10. Toxicity of propionate *in vivo*.** Levels of serum alanine aminotransferase (ALT), aspartate transaminase (AST), albumin (ALB) and total protein (TP) in the mice from different treatment groups as detected by an automated hematology analyzer. Graphs represented means ± SEM and statistical significance was calculated by one-way ANOVA. ns: no significance.

| **Table S1 Primer sequences for real-time PCR** | | | |
| --- | --- | --- | --- |
| **Gene Name** | **Species** | **Forward Sequence** | **Reverse Sequence** |
| B. fragilis | Microbiota | TCRGGAAGAAAGCTTGCT | CATCCTTTACCGGAATCCT |
| B. uniformis | Microbiota | TCTTCCGCATGGTAGAACTATTA | ACCGTGTCTCAGTTCCAATGTG |
| B. thetaiotaomicron | Microbiota | GCAAACTGGAGATGGCGA | AAGGTTTGGTGAGCCGTTA |
| B. caccae | Microbiota | AAACCCATACGCCGCAAG | GACACCTCACGGCACGAG |
| B. distasonis | Microbiota | TGCCTATCAGAGGGGGATAAC | GCAAATATTCCCATGCGGGAT |
| B. eggerthii | Microbiota | CCCGATAGTATAGTTTTTCCGC | TCCTCTCAGAACCCCTATCCAT |
| B. merdae | Microbiota | AGGGTGCGTAGGTGGTGAT | TTCACCGCTACACCACGC |
| B. splanchnicus | Microbiota | ATGTAATGATGAGCACTCTAACGG | GGCTTTTGAGATTGGCATCC |
| B. stercoris | Microbiota | GCTTGCTTTGATGGATGGC | CATGCGGGAAAACTATGCC |
| B. ovatus | Microbiota | TGCAAACTRAAGATGGC | CAAACTAATGGAACGCATC |
| B. vulgatus | Microbiota | CGGGCTTAAATTGCAGATGA | CATGCAGCACCTTCACAGAT |
| GPR41 | Human | TTCACCACCATCTATCTCACCG | GGAACTCCAGGTAGCAGGTC |
| GPR43 | Human | CCGTGCAGTACAAGCTCTCC | CTGCTCAGTCGTGTTCAAGTATT |
| ACTB | Human | CACCATTGGCAATGAGCGGTTC | AGGTCTTTGCGGATGTCCACGT |
| MYD88 | Human | GGCTGCTCTCAACATGCGA | CTGTGTCCGCACGTTCAAGA |
| NFκB | Human | AACAGAGAGGATTTCGTTTCCG | TTTGACCTGAGGGTAAGACTTCT |
| ICAM1 | Human | ATGCCCAGACATCTGTGTCC | GGGGTCTCTATGCCCAACAA |
| PTPN2 | Human | GAAGAGTTGGATACTCAGCGTC | TGCAGTTTAACACGACTGTGAT |
| TLR3 | Human | TTGCCTTGTATCTACTTTTGGGG | TCAACACTGTTATGTTTGTGGGT |
| CXCL11 | Human | GACGCTGTCTTTGCATAGGC | GGATTTAGGCATCGTTGTCCTTT |
| TNFα | Human | CCTCTCTCTAATCAGCCCTCTG | GAGGACCTGGGAGTAGATGAG |
| CCL2 | Human | CAGCCAGATGCAATCAATGCC | TGGAATCCTGAACCCACTTCT |
| IL-6 | Human | ACTCACCTCTTCAGAACGAATTG | CCATCTTTGGAAGGTTCAGGTTG |
| CXCL8 | Human | TTTTGCCAAGGAGTGCTAAAGA | AACCCTCTGCACCCAGTTTTC |
| MMP13 | Human | ACTGAGAGGCTCCGAGAAATG | GAACCCCGCATCTTGGCTT |
| CTHRC1 | Human | CAATGGCATTCCGGGTACAC | GTACACTCCGCAATTTTCCCAA |
| PDGFRB | Human | AGCACCTTCGTTCTGACCTG | TATTCTCCCGTGTCTAGCCCA |
| MMP1 | Human | AAAATTACACGCCAGATTTGCC | GGTGTGACATTACTCCAGAGTTG |
| IFIT2 | Human | AAGCACCTCAAAGGGCAAAAC | TCGGCCCATGTGATAGTAGAC |
| STAT1 | Human | CAGCTTGACTCAAAATTCCTGGA | TGAAGATTACGCTTGCTTTTCCT |
| IRF7 | Human | GCTGGACGTGACCATCATGTA | GGGCCGTATAGGAACGTGC |
| XAF1 | Human | GCTCCACGAGTCCTACTGTG | GTTCACTGCGACAGACATCTC |
| IRAK2 | Human | GAAATCAGGTGTCCCATTCCAG | TGGGGAGGTCGCTTCTCAA |
| HDAC3 | Human | CCTGGCATTGACCCATAGCC | CTCTTGGTGAAGCCTTGCATA |
| FOXK1 | Human | TCCAGGAGCCGCACTTCTA | CTCCGGGATGTGGATCTTCA |

**Table S2 Clinical characteristics of RA patients and HC individuals**

| **Variables** | **RA patients** | **Healthy controls** | **P value** |
| --- | --- | --- | --- |
| Age | 59.60 ± 8.95 | 59 ± 8.32 | ns |
| Sex (female/male) | 13/1 | 15/0 | ns |
| swollen joint counts | 6.19 ± 7.38 | NA | \ |
| tender joint counts | 8.44 ± 7.56 | NA | \ |
| ESR | 34.94 ± 17.32 | NA | \ |
| CRP | 10.76 ± 11.63 | 1.34 ± 1.26 | 0.0041 |
| DAS28-ESR | 4.89 ± 1.37 | NA | \ |

ESR, erythrocyte sedimentation rate; CRP, C-reactive protein. Statistic of the RA vs. HC: Student’s t test, two-tailed**.** ns: no significance.
